# In Silico Characterization of Glycan Ions from IM-MS Collision Cross Section

**DOI:** 10.1021/jasms.4c00370

**Published:** 2025-02-10

**Authors:** Mithony Keng, Kenneth M. Merz

**Affiliations:** Department of Chemistry, Michigan State University, East Lansing, Michigan 48824, United States; Department of Biochemistry and Molecular Biology, Michigan State University, East Lansing, Michigan 48824, United States

## Abstract

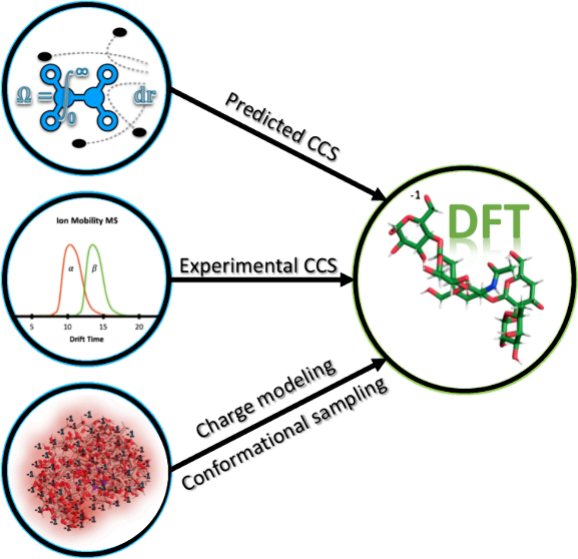

Ion mobility mass spectrometry (IM-MS) can assist in the identification of isobaric chemical analytes by exploiting the difference in their gas phase collision cross-section (CCS) property. In glycomics, reliable glycan characterization remains challenging, even with IM-MS, because of closely related isomeric species and the available binding arrangements of substituted monosaccharides, allowing for the formation of complex structures. Here, we present a computational procedure to obtain gas-phase structural information from the experimental IM-MS CCS data of carbohydrates. The workflow proceeds with high throughput charge modeling of glycan seed structures to determine the precise protonation or deprotonation site. The charge models were then screened by using density functional theory (DFT) to produce candidate charge states for conformation generation. An extensive conformational scoring of the glycan ions was performed quantum mechanically at the DFT D3-B3LYP/6-31G+(d,p) level for the negative mode, [M – H]^−^, and at the D3-B3LYP/6-31G(d,p) level for the positive mode, [M + H]^+^. For most of our test set, the computed CCS values from the final geometry optimized structures showed good agreement with experiment. We also demonstrated the capability of characterizing configurational and constitutional isomeric species. Altogether, we believe that the method we used in this work can be used to build a reliable theoretical reference database for glycans that can be used for experimental quality control and for assigning molecular structure to experimental IM-MS CCS information.

## Introduction

In almost all living organisms, carbohydrates and glycans are vital contributors to biological structure and function. Furthermore, glycoconjugates have prominently been used in healthcare to classify Lewis and ABO blood types according to their specific glycoprotein composition or antigenic polymorphisms.^1−3^ The diverse roles of glycans make them potentially invaluable and powerful novel biomarkers for the detection and monitoring of a variety of disease states.^4−9^ Therefore, to help advance diagnostic services or detection science, it is highly advantageous to have reliable computational methods to aid in the experimental characterization of targeted and nontargeted glycan structures.

Currently, the most practical and appropriate method for the qualitative identification of analytes is with mass spectrometry (MS).^10−12^ In principle, MS separates analytes based on differences in their mass-to-charge ratio (*m*/*z*); therefore, a lighter gas-phase ion of a specific charge mode (e.g., [M+nX]^+n^, [M-nX]^−n^, [M]^+^, [M]^−^) will travel faster and reach a MS detector sooner than a heavier gas phase ion of the same charge. The two commonly used methods to produce gas-phase ions are electrospray ionization (ESI) and matrix-assisted laser desorption ionization (MALDI), where MALDI typically yields singly charged molecular ions and ESI can yield singly or multicharged ion species.^13^ From the parent molecular ion, fragment legacy ions can be produced, if needed, using fragmentation techniques such as electron transfer dissociation, electron capture dissociation, electron ionization, chemical ionization, or collision induced dissociation.^14,15^ The realization of additional fragmentations is often necessary for sequencing purposes or to further differentiate between parent ions with similar or identical *m*/*z* values (i.e., isobars).

For the standard MS instrumentation that achieves separation exclusively by *m*/*z*, only fragment ions that have different masses are applicable. However, in instances where analyte ions are isobaric, which is the case for most biologically relevant glycans like stachyose, cellotetraose, maltotetraose, and mannotetraose, where they have either different types of monosaccharide compositions, different α or β glycosidic configurations (i.e., anomers), or different glycosidic connectivity (*e.g*., (1 → 2), (1 → 3), (1 → 4), and (1 → 6)) but have identical *m*/*z*, an additional physicochemical dimension must be taken into account for their successful separation.

To address these situations, ion mobility mass spectrometry (IM-MS)^16−18^ is a valuable technique for the identification of isobaric analytes by exploiting the difference in their collisional cross section (CCS).^19,20^ CCS or the rotationally averaged collision surface area is dependent only on the unique gas-phase physicochemical property of a molecule and is independent of the IM-MS instrument used. This makes CCS interlaboratory results highly reproducible given the same experimental setup or method.^21^ It should be noted that when CCS values for some arbitrarily selected glycan systems are compared between the stepped field and the calibrated single field CCS method for N_2_ (^DT^CCS_N2_) drift tube IM-MS (DTIMS), the average percent difference is 4.4% (Table 1). We hypothesize that the discrepancy between the two CCS methods, other than technical or instrument error, is due to the difference in methods used to obtain the CCS value from the analyte ion arrival time. While the stepped field method performs linear regression to determine the arrival time of ions by varying the electric field strengths, the single field method utilizes a uniform low electric field and regression analysis of the calibrant ions.^22^ CCS values can then be derived from arrival time data using the Mason-Schamp equation.^23^

**Table 1:** Glycans Reference ^DT^CCS_N2_ Comparison for the DTIMS Stepped Field and Calibrated Single Field Method^a^

Glycans	Charge Mode	Stepped Field CCS^42^ (Å^2^)	Single Field CCS^43,44^ (Å^2^)	|Δ| CCS	****%Δ***C***CS****
Cellobiose	[M − H]^−^	167.86	179.70	11.84	6.81
Glucosamine	[M + H]^+^	135.59	139.75 ± 2.3	4.16	3.02
Glucuronate	[M − H]^−^	134.23	131.70	2.53	1.90
Inositol	[M − H]^−^	144.27	129.96 ± 1.9	14.32	10.44
Isomaltose	[M − H]^−^	180.77	171.96	8.81	5.00
Lactose	[M − H]^−^	170.23	176.90	6.67	3.84
Maltose	[M − H]^−^	205.90	180.10	25.80	13.37
Palatinose	[M − H]^−^	172.60	177.90	5.30	3.02
Raffinose	[M − H]^−^	197.59	197.30	0.29	0.15
Stachyose	[M − H]^−^	226.67	222.61 ± 2	4.06	1.81
Sucrose	[M − H]^−^	168.47	168.20	0.27	0.16
Xylitol	[M − H]^−^	127.92	124.1 ± 1.7	3.82	3.03
Average					4.4 ± 4

a Values for each method are averaged when applicable. The percent difference, %Δ, is used to quantify the degree of discrepancy between the two experimental CCS determination methods.

In their work on DeepCCS,^24^ Plante et al. discovered calculated reference CCS errors for methyl behenate, l-threonine, and d-maltose anions. For their repeated IM-MS experiment they obtained an average CCS of 168.8 Å^2^ for maltose, which ultimately lead them to conclude that the stepped field CCS reference of 205.9 Å^2^ for maltose had originated from aggregated ions. We note that for analytes that have CCS results from multiple IM-MS sources, misinterpretation of the results is discoverable; however, when a single experimental source is available, which is a common occurrence, then no comparison of data can be made, and it is nearly impossible to detect or rule out misinterpreted experimental results. In view of this, it is desirable to have access to both theoretical CCS reference values and the corresponding gas-phase structures as tools to aid in quality control.

With the growing need to expand the services provided by MS experiments in both academia and industry, the capability to assign structural information with molecular-level detail directly from IM-MS data is increasingly necessary. To the best of our knowledge, there is currently no high-throughput computational method for reliable glycan gas-phase structure assignment.^25,26^ This is because the challenge with modeling glycans compared to other biomolecule classes is that monosaccharide residues are composed of numerous hydroxyl groups (∼5 OH per monosaccharide residue) that are capable of harboring charges in an ESI solvent. The close vicinity between the OH groups also create an environment conducive for charge/proton migration along the direction of relatively strong intramolecular interactions such as hydrogen bonding in the gas phase after ESI.^27,28^ Moreover, finding the location of the charge bearing site on a glycan ion becomes increasingly time-consuming and more computationally expensive with each additional monomer residue added. Nevertheless, determining the correct charge state is crucial because the location of the charge can significantly influence an ion’s conformational size and shape.

**Figure 1:**
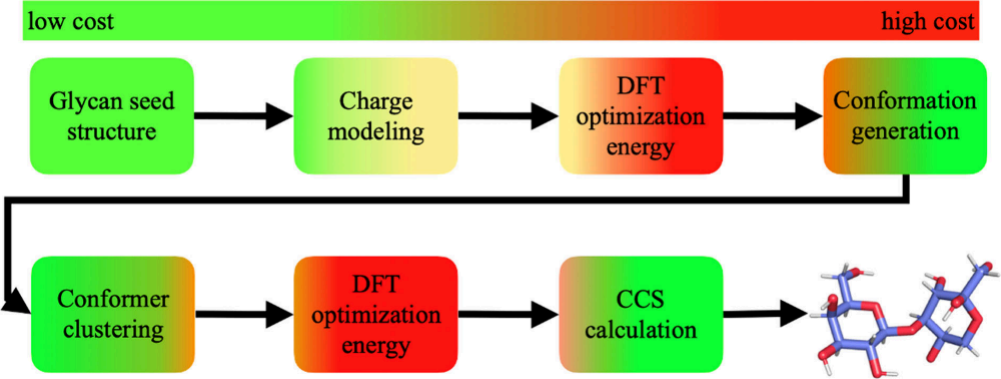
Steps implemented in this work for assigning experimental gas-phase molecular ion structures. The color gradient indicates the relative degree of computational cost incurred for each step.

Glycosidic bonds have a high energy barrier to rotation about the reducing acetal carbon, and in a typical IM-MS condition, the α or β glycosidic configuration is locked in. The fact that glycans have few rotatable bonds relative to their overall molecular size is a characteristic that we will exploit to forego a conformational ensemble for the initial charge state determination step, and in doing so, we can greatly speed up the overall modeling task. Here, we present an in-silico protocol to accurately resolve glycan gas phase structures with reasonable efficiency. The workflow that we use is adopted from our established POMICS^29^ workflow, which we have used to annotate small molecule metabolites in the gas-phase. The steps include high-throughput charge evaluation, extensive conformational sampling, and the use of density functional theory (DFT) for geometry optimization and relative energy (RE) scoring (Figure 1). Furthermore, we use the reference CCS values to both guide the structure assignment and as a performance metric, which means that for an assignment to be deemed “successful” it must achieve a computed CCS error of ≤3%, which we validated earlier for standard metabolites.^29^ We use the 3% margin of error^29^ to account for the average instrument calibration error and for the observed uncertainty in experimental IM-MS results across different instruments and sites.

## Computational Methods

### Seed Generation and Charge Modeling

The initial 3D glycan starting structures were either obtained from the Glycam Web server or from the RCSB PDB database. Glycam built structures were automatically refined using molecular dynamics simulations with the GLYCAM06^30^ force field. Chemical group addition to GLYCAM structures to allow for the modeling of derivatized glycans were completed using PyMOL.^31^ Systems that were not built successfully through Glycam (e.g., sugar alcohol, cyclic glycans, unsupported compositional isomers) were obtained from the RCSB database. A list of RCSB sources can be found in Table S1 in the Supporting Information. Unlike Glycam produced structures, RCSB seed structures were not refined using a force field, but entered the charge modeling step in their default bioactive conformation.

To carry out a high-throughput charge modeling campaign, an in-house python program was used to cycle through titratable sites by systematically deprotonating or protonating the seed structures to produce the negative mode (i.e., [M − H]^−^) or positive mode (*i.e*., [M + H]^+^) charge models. All probable acidic or basic sites (e.g., carbonyl, hydroxyl, and amine) on the glycan seed structures were sampled to generate viable charge models. Executing a thorough sampling of possible charge models is necessary because even though there are programs and methods available for p*K*_a_ (solution phase acidity) estimation,^32−34^ their reliability for locating the protonation or deprotonation site on an isolated molecule in the gas-phase has not been established, therefore, it is a best practice approach to survey the potential gas-phase carbohydrate protonation states quantum mechanically.^35−37^

### DFT Candidate Charge Model Scoring

All quantum mechanical (QM) calculations were completed using the Gaussian16 software package.^38^ To find the most stable gas phase charge site from the glycan charge models, D3BJ-B3LYP/6-31G(d,p) and D3BJ-B3LYP/6-31+G(d,p) were utilized for positive ion mode and negative ion mode, respectively. To maintain consistency, the same DFT level of theory was employed for both geometry optimization and charge calculations. The charge model for each glycan system that produced the lowest relative electronic energy (*RE*) was selected as the primary candidate charge state for conformation generation. In addition, any charge model that showed an electronic energy within 10 kcal mol^–1^ of its respective global energy minimum charge state candidate (*RE* = 0 kcal mol^–1^) was also selected as a charge state for conformation generation.

### Conformation Generation and Clustering

ConfGen^39^ was used to generate conformations for each candidate charge state. Ideally, we set the number of conformations to be generated to 1000; however, for some systems 1000 conformations were not possible due to the low degrees of freedom for the molecule. To significantly reduce the computational cost of running the standard 1000 conformers for each charge state at the DFT level of theory, all charge states with more than 50 generated conformers were clustered using the open-source software AutoGraph.^40^ The resulting representative conformer centers generated by AutoGraph clustering were carried forward to the DFT geometry optimization step.

### Electronic Energy and CCS Calculations

Geometry optimization and single-point energy calculations for the candidate charge state ensembles were performed at the DFT D3BJ-B3LYP/6-31+G(d,p) level of theory for singly charged negative mode ions, [M − H]^−^. For the singly charged positive mode ions, [M + H]^+^, the gas-phase geometry optimization and single-point spectroscopy were obtained at the D3BJ-B3LYP/6-31G(d,p) level. The Mulliken charge scheme was used as the default charge scheme in Gaussian16 for atomic partial charge calculation for all glycan systems.

The CCS values for the DFT optimized glycan candidate structures were computed using the high performance collision cross section^41^ (HPCCS) package. HPCCS was developed with atomic partial charges generated by using the Mulliken charges. The program takes in the molecular partial charges and atomic positions produced from the DFT optimization step as inputs, and from there it calculates the CCS using the trajectory method.^42^ The parameters for HPCCS were set as follows: 10 for the number of complete cycles; 20 for velocity integration; 500 for Monte Carlo integrations; 1000 for the number of rotations; the simulation temperature was set to 298 K; nitrogen was set as the buffer gas.

### Candidate Structure Selection

The candidate structures from the representative ensembles for all glycan systems were scored according to their RE. The final selected gas-phase conformation was the global minimum geometry of the system. Since there may be more than one low energy conformer per system that is populated in the gas-phase, we used the Boltzmann distribution as a function of energy (*i.e*., *RE*) to calculate the mole fraction (*i.e*., probability) of the low energy conformer(s) (eqs 1 and 2).
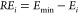
1
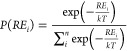
2where *RE*_*i*_, *E*_*i*_, and *E*_*min*_ are the relative energy, absolute energy (i.e., electronic energy), and minimum absolute energy (*i.e*., global minimum conformer) for a system. *P* is the probability (*i.e*., mole fraction) of finding a system in a conformational state with *RE*_*i*_ that is normalized to the total number (*n*) of conformers sampled. *k* and *T* are the Boltzmann constant and absolute temperature, which was set to 298 K. So, for systems that have conformers that are within approximately 3 kcal mol^–1^ of their respective global minimum conformer (*RE* = 0 kcal mol^–1^) the probability of having more than one conformer populated is >0%, which we accounted for by reporting the weighted-average computed CCS values (see Table S2 in the Supporting Information).

It should be noted that CCS agreement alone to assign the experimental charge state and conformation should be carefully considered since CCS only gives information about an ion’s size and overall shape. Additionally, the CCS space may be broad and can often encapsulate several chemically distinct ions due to similarities in size and similar energies. On the other hand, theoretical QM structure assignment requires searching a conformational space to arrive at an energy minimum geometry. Thus, the combination of achieving a low energy conformation (*i.e*., global minimum) and a good CCS agreement (*i.e*., ≤3% error) allow for the reasonable assignment of experimentally viable conformations despite this apparent limitation.

### Experimental CCS Reference Source

In the present work, we only use reference CCS values obtained from DTIMS experiments and not from TWIMS because there are more data currently available for the former pertaining to glycan systems that are relevant to this work. The reference CCS values for glycans were procured and averaged for the stepped field^43^ experiment or the single field^44,45^ experiments when multiple data exist.

## Results and Discussion

In what follows, we explored two approaches for glycan structure prediction based on CCS values. In the first “fast” approach, we deprotonated the experimental or GYCAM derived structure, and energy minimized the resultant structure followed by CCS determination. The reason for choosing this approach was to obtain a baseline for an approach that requires minimal computational capabilities. The second “slow” approach involved the generation of a conformational ensemble using a conformational search protocol and is computationally more expensive due to the need to do many more QM calculations relative to the baseline approach.

### Glycan Structure Assignment without Conformational Sampling

**Figure 2:**
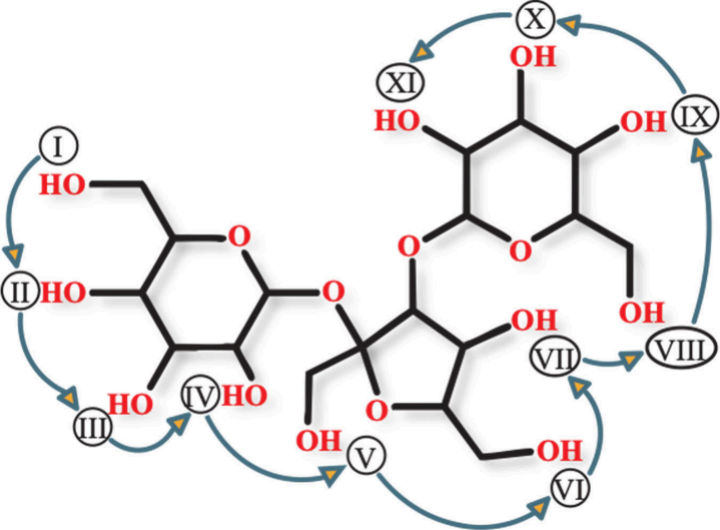
Charge modeling scheme used for finding candidate charge states, as exemplified with melezitose. Arrows indicate enumeration of titratable sites for the protonated form, [M + H]^+^, or deprotonated form, [M – H]^−^, to locate low energy minimum charge models. For simplicity, only enumerated hydroxyl sites are shown here.

Figure 2 shows an example of titratable or charge site enumeration for negative and positive modes to locate low energy charge models (*e.g*., DFT relative energy of ≤10 kcal mol^–1^ of the global minimum energy charge model) for the conformation generation step. In this analysis we systematically go from one ionizable position to another (I → II → ... XI) and add or delete a proton to generate the negative or positive mode candidate structures. For a complete structural illustration of the final determined charge states, see Figure S1 in the Supporting Information.

**Figure 3:**
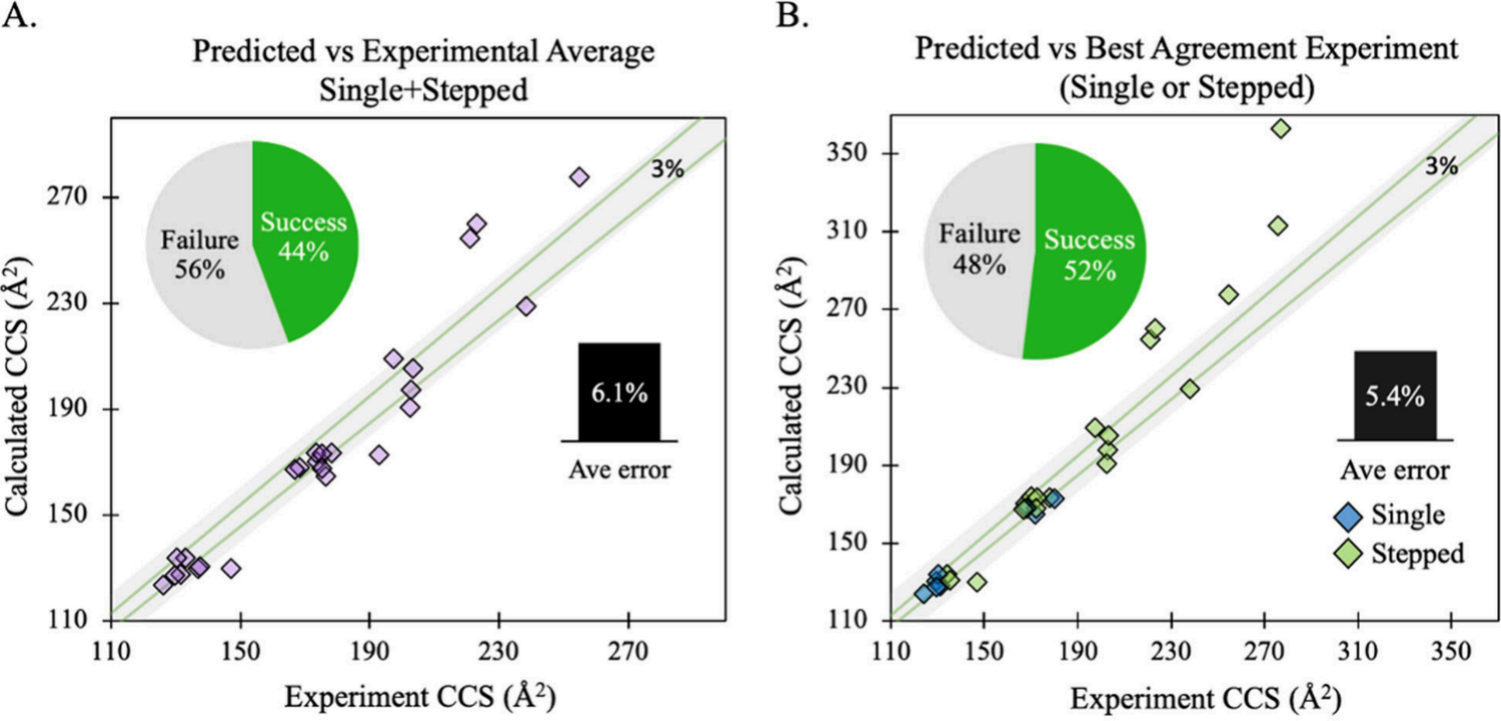
Structure assignment success rate and accuracy results without conformational ensemble sampling. (A) Performance of predicted CCS compared to CCS produced from averaging single and stepped field experiments. (B) Performance of predicted CCS compared to experimental CCS from either single (blue diamond) or stepped field (green diamond) method that has the better agreement with prediction (lower error). The gray region between the green diagonal lines represents the 3% error threshold. The number of predicted systems that fall within this gray region (≤3%) determines the structure assignment success rate.

Before we proceeded to conformation ensemble generation for the determined candidate charge states, we first evaluated the CCS for the DFT optimized structures with the experimental reference using the “fast” approach. The computed CCS performance results for the assigned structures before conformational sampling are shown in Table 2. When we evaluate the performance at the level of the two different CCS experimental methods, the structure assignment success rate is ∼53% (∼3% error) and ∼42% (∼7% error) for the single field and stepped field, respectively. The average % error for the assigned glycan ions when compared to the average of the single field and stepped field (⟨single + stepped⟩) CCS experiment is ∼6%. Moreover, the structure assignment success rate versus ⟨single + stepped⟩ is ∼44% (12 out of 27 systems achieved ≤3% CCS error) (Figure 3A) or ∼52% when compared only with the method (single or stepped) that gives the better CCS agreement on a system-to-system basis (Figure 3B). Accordingly, the results indicate that we have a reasonable hypothesis of the charge states for nearly half of the systems tested by using the “fast” approach. Among the systems with ≥4 monomer residues (*i.e*., lacto-N-fucopentaose I, lacto-N-neotetraose, maltotetraose, mannohexaose, and mannotetraose), the average error is ∼17%, which indicates that the charge states determined may be incorrect and/or that their conformational spaces are large enough to be insufficiently represented by a single conformer. Although the rigidity of glycosidic bonds persists regardless of the glycan size, the conformational space overall increases with an increasing number of monomer residues.

**Table 2:** Computed CCS Values for the Glycan Charge Model Candidates (Pre conformation Sampling)^a^

	Glycan	Charge Mode	Cal. CCS (Å^2^)	Single Field CCS (Å^2^)^c^	Stepped Field CCS (Å^2^)^c^	⟨Single+Stepped⟩ CCS (Å^2^)^b^^c^
1	Cellobiose	[M − H]^−^	170	179.70(5.38)	167.86(1.29)	173.78(2.16)
2	Glucuronate	[M − H]^−^	134	131.70(1.53)	134.23(0.38)	132.97(0.57)
3	Inositol	[M − H]^−^	130	129.95(0.03)	144.27(9.95)	137.11(5.25)
4	Isomaltose	[M − H]^−^	164.49	171.96(4.34)	180.77(9.01)	176.37(6.73)
5	Isomaltotriose	[M − H]^−^	197.44	^—^	202.92(2.70)	202.92(2.70)
6	Lacto-N-Fucopentaose I	[M − H]^−^	312.79	^—^	275.87(13.38)	275.87(13.38)
7	Lacto-N-Neotetraose	[M − H]^−^	277.59	^—^	254.73(8.97)	254.73(8.97)
8	Lactose	[M − H]^−^	173.41	176.90(1.97)	170.23(1.87)	173.57(0.09)
9	Lactulose	[M − H]^−^	173.30	^—^	178.23(2.77)	178.23(2.77)
10	Maltose	[M − H]^−^	173	180.10(4.19)	205.90(16.20)	193.00(10.60)
11	Maltotetraose	[M − H]^−^	254.38	^—^	221.08(15.06)	221.08(15.06)
12	Mannitol	[M − H]^−^	127.44	131.60(3.16)	^—^	131.60(3.16)
13	Mannohexaose	[M − H]^−^	363.03	^—^	277.27(30.93)	277.27(30.93)
14	Mannotetraose	[M − H]^−^	259.88	^—^	223.26(16.40)	223.26(16.40)
15	Melezitose	[M − H]^−^	205.21	^—^	203.52(0.83)	203.52(0.83)
16	Melibiose	[M − H]^−^	173	178.10(2.93)	172.63(0.14)	175.36(1.42)
17	Palatinose	[M − H]^−^	168	177.90(5.76)	172.60(2.87)	175.25(4.34)
18	Raffinose	[M − H]^−^	209	197.30(6.00)	197.59(5.85)	197.45(5.92)
19	Sorbitol	[M − H]^−^	133.72	130.52(2.45)	^—^	130.52(2.45)
20	Sucrose	[M − H]^−^	167.8	168.20(0.24)	168.47(0.40)	168.34(0.32)
21	Tagatose	[M − H]^−^	127.00	129.77(2.13)	^—^	129.77(2.13)
22	Xylitol	[M − H]^−^	124	124.33(0.76)	127.92(3.54)	126.13(2.17)
23	Xylobiose	[M − H]^−^	167.29	^—^	166.93(0.22)	166.93(0.22)
24	Glucosamine	[M + H]^+^	131	139.75(6.52)	135.59(3.65)	137.67(5.11)
25	Maltotetraose	[M + H]^+^	228.95	^—^	238.30(3.92)	238.30(3.92)
26	Melezitose	[M + H]^+^	190.63	^—^	202.60(5.91)	202.60(5.91)
27	Sorbitol	[M + H]^+^	129.83	^—^	147.20(11.80)	147.20(11.80)
	Average % error			(3 ± 2)	(7 ± 7)	(6 ± 7)

a The negative and positive mode candidate structures were geometry optimized at the D3BJ-B3LYP/6-31+G(d,p) and B3LYP/6-31G(d,p) level, respectively. The entry (−) in the single or stepped field column indicates no experimental value available for that system.

b Average of available reference CCS values from stepped field and single field methods.

c Value in parentheses is percent CCS error between calculated CCS and reference CCS.

### Structure Assignment Performance with Conformational Sampling

**Figure 4:**
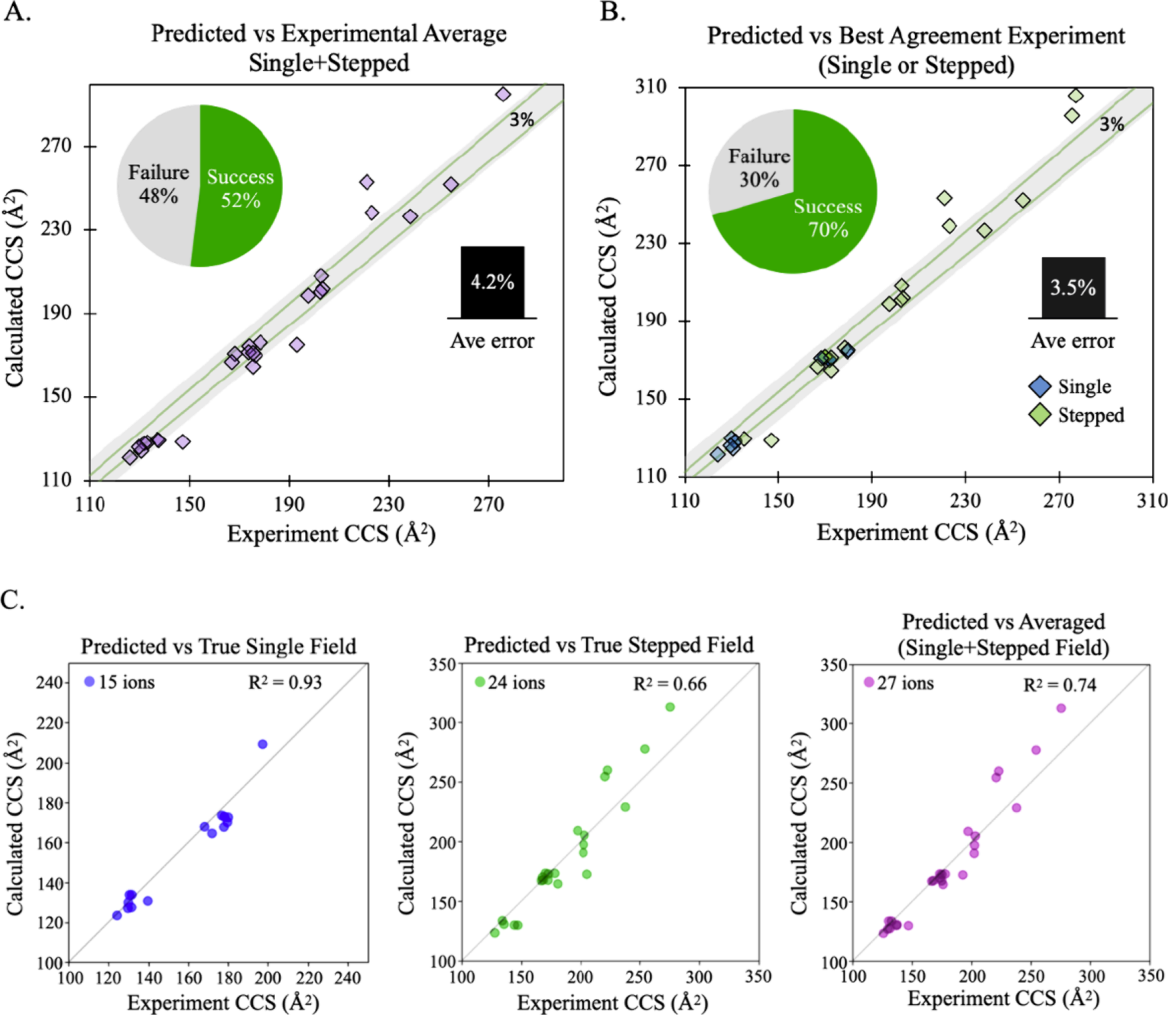
Structure assignment success rate and accuracy results after conformational ensemble sampling. (A) Prediction accuracy compared to the average of CCS values from single and stepped field experiments. (B) Prediction accuracy compared to CCS from single (blue diamond) or stepped field (green diamond) that has the better agreement with prediction. (C) Correlation (R^2^) values showing prediction accuracy conditionally depend on the experimental method used as reference.

When we include ensemble sampling for the candidate charge state ions (for a complete assignment of the relative energies of the conformational ensemble for the various charge states, see Table S3 in the Supporting Information**)**, the average CCS error for ⟨single + stepped⟩ improved to ∼4% (Table 3) and the standard deviation is reduced to ±4% from ±7%. Figure 4A shows that although the average CCS error is still above our ideal target of 3%, the structure assignment success rate increases to ∼52% versus ⟨single + stepped⟩ or ∼70% when compared to the method (single or stepped) that gives the better CCS agreement on a system-to-system basis (Figure 4B). Additionally, the accuracy of the assigned structures is conditionally dependent on the reference type used for comparison, as demonstrated by the different R^2^ values shown in Figure 4C. For this work, we find that the single field method shows the best correlation (*i.e*., R^2^ = 0.93) with our computed CCS values, although the sample size is smaller than for the stepped field method.

**Table 3:** Computed CCS Values for the Glycan Charge Model Candidates (Post-conformation Sampling)^a^

	Glycan	Charge Mode	Cal. CCS (Å^2^)	Single Field CCS (Å^2^)	Stepped Field CCS (Å^2^)	⟨Single+Stepped⟩^a^ CCS (Å^2^)
1	Cellobiose	[M − H]^−^	174.33	179.70(2.99)	167.86(3.85)	173.78(0.32)
2	Glucuronate	[M − H]^−^	128	131.70(2.50)	134.23(4.34)	132.97(3.43)
3	Inositol	[M − H]^−^	130	129.95(0.14)	144.27(10.05)	137.11(5.36)
4	Isomaltose	[M − H]^−^	170	171.96(1.10)	180.77(5.92)	176.37(3.57)
5	Isomaltotriose	[M − H]^−^	207.99	^—^	202.92(2.50)	202.92(2.50)
6	Lacto-N-Fucopentaose I	[M − H]^−^	295.35	^—^	275.87(7.06)	275.87(7.06)
7	Lacto-N-Neotetraose	[M − H]^−^	251.96	^—^	254.73(1.09)	254.73(1.09)
8	Lactose	[M − H]^−^	172	176.90(3.05)	170.23(0.75)	173.57(1.18)
9	Lactulose	[M − H]^−^	176.12	^—^	178.23(1.18)	178.23(1.18)
10	Maltose	[M − H]^−^	175.19	180.10(2.73)	205.90(14.92)	193.00(9.23)
11	Maltotetraose	[M − H]^−^	253.18	^—^	221.08(14.52)	221.08(14.52)
12	Mannitol	[M − H]^−^	127.93	131.60(2.79)	^—^	131.60(2.79)
13	Mannohexaose	[M − H]^−^	305.88	^—^	277.27(14.52)	277.27(10.32)
14	Mannotetraose	[M − H]^−^	238.53	^—^	223.26(6.84)	223.26(6.84)
15	Melezitose	[M − H]^−^	201.76	^—^	203.52(0.86)	203.52(0.86)
16	Melibiose	[M − H]^−^	165	178.10(7.48)	172.63(4.55)	175.36(6.04)
17	Palatinose	[M − H]^−^	171	177.90(3.74)	172.60(0.79)	175.25(2.29)
18	Raffinose	[M − H]^−^	198.7	197.30(0.68)	197.59(0.54)	197.45(0.61)
19	Sorbitol	[M − H]^−^	124.41	130.52(4.68)	^—^	130.52(4.68)
20	Sucrose	[M − H]^−^	171	168.20(1.49)	168.47(1.33)	168.34(1.41)
21	Tagatose	[M − H]^−^	126.15	129.77(2.79)	^—^	129.77(2.79)
22	Xylitol	[M − H]^−^	121	124.33(2.48)	127.92(5.21)	126.13(3.87)
23	Xylobiose	[M − H]^−^	166.75	^—^	166.93(0.11)	166.93(0.11)
24	Glucosamine	[M + H]^+^	129	139.75(7.50)	135.59(4.66)	137.67(6.10)
25	Maltotetraose	[M + H]^+^	236.51	^—^	238.30(0.75)	238.30(0.75)
26	Melezitose	[M + H]^+^	200.53	^—^	202.60(1.02)	202.60(1.02)
27	Sorbitol	[M + H]^+^	128.66	^—^	147.20(12.60)	147.20(12.60)
	Average % error			(3 ± 2)	(5 ± 5)	(4 ± 4)

a The negative and positive mode candidate structures were geometry optimized at the D3BJ-B3LYP/6-31+G(d,p) and B3LYP/6-31G(d,p) level, respectively. The entry (−) in the single or stepped field column indicates no experimental value available for that system.

As expected, conformational sampling improves the match between theory and experiment though at a significant increase in computational expense since more conformations required us to perform ∼6-fold more DFT optimizations for the “slow” compared to the “fast” approach. For example, lacto-N-neotetraose achieves the most impressive improvement in accuracy going from an error of ∼9% to ∼1% after conformational sampling. It should be noted that ∼70% of the time either the single field or stepped field CCS value agrees with our computed CCS within a 3% error. With that said, when considering only the reference method that best agrees with our assignment, the average CCS error improved to ∼3.5%. This is meaningful since it demonstrates that we can use our theoretical CCS values to assist in quality control or validate IM-MS experiments, especially in the case where there is ambiguity in analyte identification due to inconsistency in results between competing CCS determination methods.

In general, we believe that the number of conformers that were generated per glycan ion system is sufficient to account for the IM-MS relevant conformational space; therefore, the continued poor performance could be due to the incorrect charge states being modeled, especially for systems that produce very large CCS errors. For example, to help achieve the correct charge state for glycans with ≥4 monomer residues, the relative energy limit could be increased or, albeit more time-consuming, exhaustively sampling all possible charge sites. Regardless, the computed outcomes achieved herein are quite good without adding this extra step.

**Figure 5:**
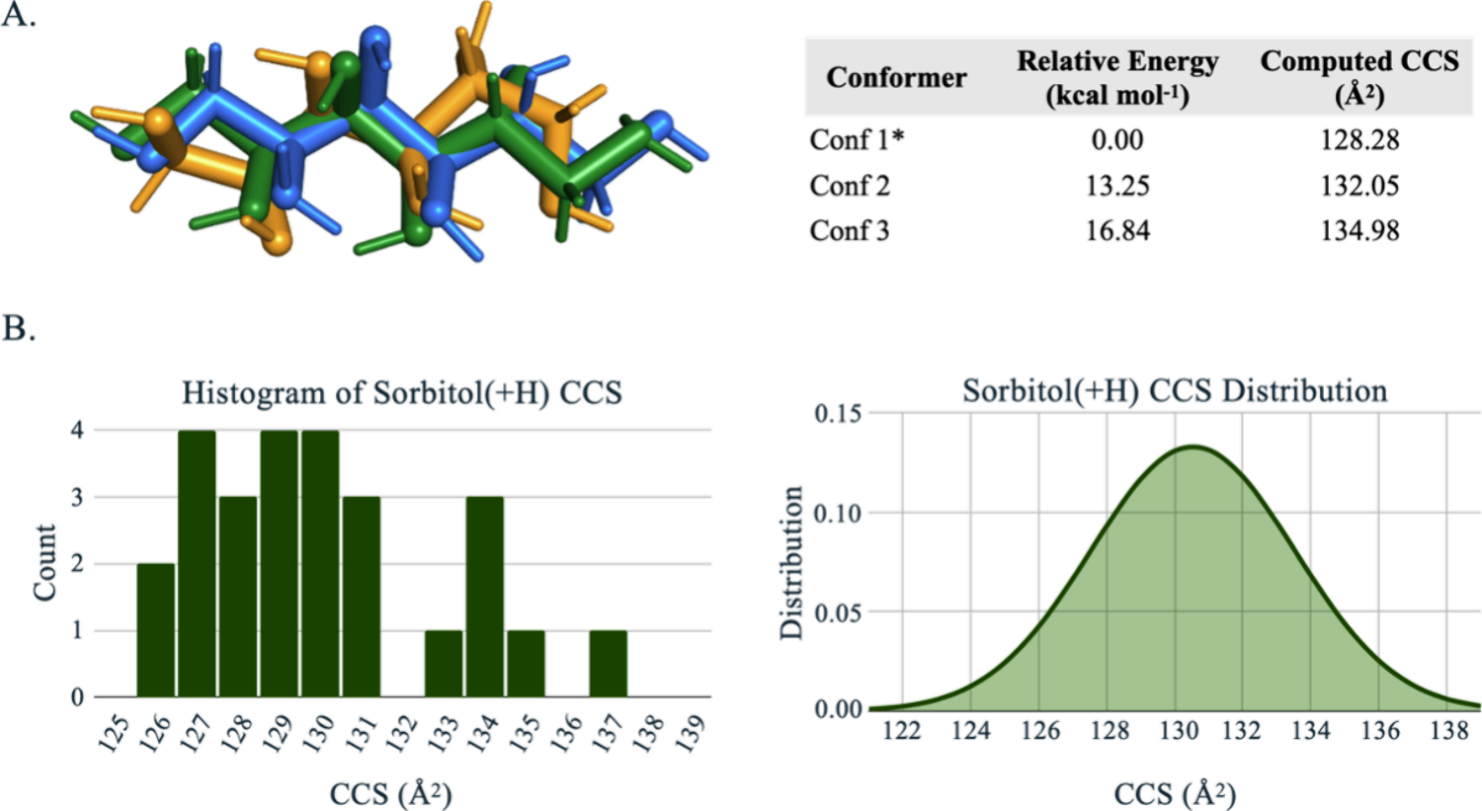
Sorbitol(+H) outlier evaluation results. (A) Optimized sorbitol(+H) candidate charge state structure (Conf 1*: orange), sorbitol(+H) extended structure with an internal OH site protonated (Conf 2: blue), and sorbitol(+H) extended structure with a terminal OH site protonated (Conf 3: green). (B) CCS distribution curve of 26 conformer centers for the sorbitol(+H) candidate charge state. Note: the reference stepped field experimental CCS value is 147.2 Å^2^.

Interestingly, the final candidate structure for sorbitol(+H) shows very poor agreement with experiment despite having fewer or similar number of rotatable bonds as other glycans with much better performance. For example, sorbitol and mannitol are isomers that only differ in the orientation of the OH group at the C2 position, however, the CCS error for mannitol(-H) is ∼2.8% compared to ∼4.7% for sorbitol(-H) and 12.6% for sorbitol(+H). We also evaluated two additional sorbitol(+H) charge states, that have extended conformations in an attempt to expose more collision surface. However, they have electronic energies >10 kcal mol^–1^ above the global minimum structure. For these cases we found that their computed CCS values are still significantly smaller than the experiment (*e.g*., > 8% error) (Figure 5A). Moreover, we observe no conformer states that were generated by Autograph for sorbitol(+H) that produce CCS distributions near the experimental CCS (Figure 5B), which is again surprising because the conformational space of sorbitol is relatively limited compared with other glycans that we have tested for this work. The conformational flexibility of sorbitol in solution and vacuum was studied by Lerbret et al., and their results showed that the dominant populations for both environments are limited to mainly eight conformers.^46^ All things considered, for an isolated sorbitol(+H) ion, we rationalize that there is not enough averaged collision surface to generate the much larger CCS that is given by the reference. With that said, we hypothesize that the root cause for the observed deviation from the CCS reference may be due to a poor fitting of sorbitol(+H) (i.e., outlier) to the stepped field regression curve, ion-neutral clustering/declustering^47^ effect, or an incident of ion aggregation as reported in the work of Plante et al.

### Characterization of Isomeric Glycans

We have thus far demonstrated the capability to assign to IM-MS CCS a finite number of candidate conformations that are, in most cases, in good agreement with experiment. The focus now is to evaluate the sensitivity of our method to faithfully represent isomeric glycans that have different configurational, constitutional, and compositional characteristics. For this task, we used the experimental work of Hofmann et al. where they studied six derivatized glycan isomers composed of a glucose and/or galactose residue with an aminoalkyl linker attached at the reducing-end.^48^ In their work, the authors reported that configurational isomers (*i.e*., α and β anomers) and constitutional isomers (*i.e*., 1 → 3 and 1 → 4 linkage) can be successfully separated using IM-MS, however, the arrival times for compositional isomers or epimers are indistinguishable since their conformational sizes in the gas-phase are nearly identical. Additionally, the authors reported that the β anomer and 1 → 3 constitutional isomer for their derivatized glycans have a larger CCS than the α anomer and 1 → 4 isomer, respectively. Similarly, our survey of the molecular surface area (MSA) for 36 unique α and β pairs of disaccharides generated using Glycam show that the β anomers are on average ∼2% larger than the α (see Table S4 in the Supporting Information). Also consistent with this observation is that we observe larger CCS values for the 1 → 3 glycosidic linkage than for the 1 → 4 linkage.

Figure S2 in the Supporting Information shows the structure and the charge site for the lowest energy structure (*i.e*., *RE* = 0 kcal mol^–1^) for each of the six derivatized glycan isomers after the initial DFT optimization and single point energy calculation. Moreover, *isomer 5* produced only one charge state, whereas the other isomers (*1*, *2*, *3*, *4*, and *6*) yield two to three viable candidate charge states that have *RE* values within 10 kcal mol^–1^ of their respective energy minimum charge state (see Table S5 in the Supporting Information**)**. We report that overall, the Boltzmann-weighted average CCS values (Table 4) for the candidate charge states (pre conformational sampling using the “fast” approach) have very poor agreement with experiment, with an average CCS error of ∼14.6%. The suboptimal performance is not surprising, since the aminoalkyl tail is highly flexible. At this stage, we do not observe the isomeric trends in CCS values that were reported by Hofmann et al. (2015).

**Table 4:**
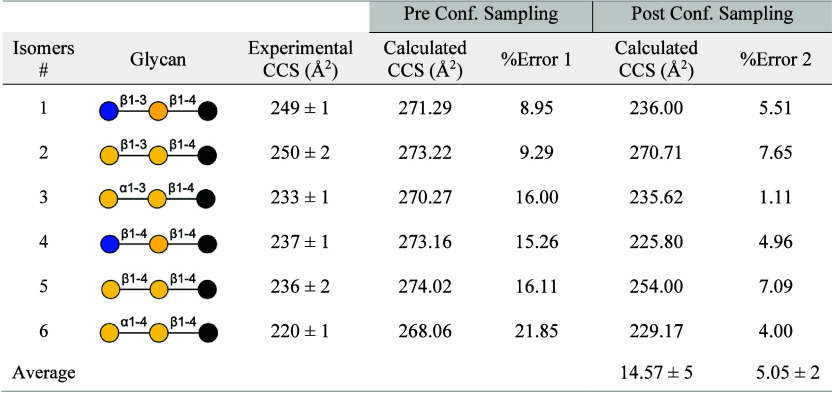
Computed CCS Results for Six Derivatized Glycan Isomers Prior to and after Conformation Sampling^a^

a Constitutional isomers are pairs *1* & *4*, *2* & *5*, or *3* & *6*. Epimers are isomer pairs *1* & *2* or *4* & *5*. Anomers are isomer pairs *2* & *3* or *5* & *6*. Blue, yellow, black circles are glucose, galactose, and amino alkyl esterified glucose at the C1 position, respectively.

After conformational sampling (i.e., 1000 conformers) the average CCS error improved to approximately 5%, with *isomer 3* achieving the best assignment accuracy at ∼1% error. Notably, we now observe CCS trends that are characteristic of the different isomeric species reported by Hofmann et al.; for each isomer pair (numbered 1–6) of an epimer type (galactose or glucose) having the same glycosidic linkage (1 → 3 or 1 → 4), the β anomer has the larger CCS relative to the α anomer (e.g., *isomer 2* > 3, and 5 > 6); similarly, for each isomer pair (1–6) of an epimer type or of an α or β anomer, the one containing the 1 → 3 linkage has the larger CCS than the 1 → 4 linkage (e.g., *isomer 1* > 4, 2 > 5, and 3 > 6). We also observe a difference in CCS between epimers (*isomer 1* and 2), but this difference is within the error bars of our computational tools.

Though we believe that the initial 1000 conformers are sufficient for handling the derivatized isomers, as exemplified by the good accuracy for *isomer 3*, we went forward and tested an ensemble size of 3000 conformers for *isomer 1*. The Boltzmann’s weighted CCS for 29 conformer centers (produced from Autograph clustering) for *isomer 1* is 263.9 Å^2^ (∼5.6% error), which is an overestimation of the experimental CCS compared to an underestimation (236.0 Å^2^) when 1000 conformers were used. Despite the larger ensemble size, the lowest energy structure was identical in both cases.

From our experience, differences in conformational size or structural compactness after DFT geometry optimization can also be attributed to the choice of damping functions implemented for the D3 dispersion correction. Using the conformer centers for *isomer 1* from the initial clustering output, we switched from an attractive close-range behavior of D3BJ to D30 (*e.g*., D3 with the zero damping function), which, in contrast, is more repulsive at close interatomic distances.^49^ We hypothesized that this would lead to a less compact structure, which might better align with experiment. Interestingly, the result for the CCS obtained using D3(0)-B3LYP/6-31+G(d,p) is ∼235 Å^2^ at a ∼ 6% error, which is nearly identical to the CCS computed for the same *isomer 1* charge model using D3BJ. Overall, we have largely eliminated both ensemble size and the degree of short-range attraction as sources of the assignment errors.

## Conclusion

Collision cross section is a useful gas-phase molecular descriptor that has been successfully and increasingly utilized in mass spectrometry for the separation of target analyte ions with similar or identical *m*/*z*. However, the lack of available methods for accurately resolving molecular-level details from experimentally derived CCS data has not helped to improve the range of services and potential applicability of IM-MS results. In this work, we have provided an efficient and effective protocol that synergistically incorporates molecular modeling, quantum mechanical calculations, and CCS assessment to generate reasonably accurate low energy gas-phase structures. Moreover, we show that our in-silico method can achieve an overall average assignment error of <3% for glycans with less than four monomer residues. Noteworthy, is that a majority (*i.e*., ∼70%) of the final structures that we have assigned have computed CCS values that agrees to within a 3% error of the CCS values obtained using either the stepped field or single field method for DTIMS. In this work, we show that CCS calculations from the single field method align better with our theoretical results.

Finally, a challenging test utilizing glycan derivatives demonstrated that we can, with reasonable precision, account for and represent faithfully the anomeric and epimeric CCS characteristics that have been documented experimentally, albeit the overall predicted CCS agreement with experiment is suboptimal, which we believe may be attributed to the conformation generator used (*i.e*., ConfGen) or the inadequacy of our modeling approach to accurately locate the charge state for highly flexible glycans. Nevertheless, we demonstrated that the procedure implemented for this work can be used, for example, to efficiently build a reliable glycan theoretical reference database for experimental quality control and/or for assigning valid molecular structure to IM-MS CCS values.

## Supporting Information Available

Test systems assigned charge sites, DFT relative energies for charge models, DFT relative energies for conformation ensembles, and molecular surface area measurements for α and β isomers (PDF)
