# Supplementary material for: In Silico Characterization of Glycan Ions from IM-MS Collision Cross Section
Source: J Am Soc Mass Spectrom. 2025 Feb 10;36(3):504–13. doi: 10.1021/jasms.4c00370 (PMC11887428; doi:10.1021/jasms.4c00370)
Supplement: Supplementary file 1 — js4c00370_si_001.pdf [file js4c00370_si_001.pdf]

Supporting Information

In Silico Characterization of Glycan Ions from IM-MS Collision Cross Section

Mithony Keng and Kenneth M. Merz, Jr.*

Department of Chemistry, Michigan State University,

East Lansing, Michigan 48824, United States

Department of Biochemistry and Molecular Biology, Michigan State University,

East Lansing, Michigan 48824, United States

*Corresponding Author: Kenneth M. Merz

*Corresponding Author Email: merz@chemistry.msu.edu

Table S1. List of rcsb PDB sources for glycan crystallographic seed structures used.

Glycan	PDB Identifier
Glucosamine	4rya
Glucuronic acid	3aon
Inositol	1awb
Mannitol	6prg
Raffinose	3alu
Sorbitol	2vft
Xylitol	7dfk

Table S2. Candidate charge states results using a single conformer per charge madel.

Glycan	[M-H]- Charge State	Cal. CCS (Å^2)	Relative Energy (kcal/mol)
Isomaltose			
1	Charge model 5	164.40	0.00
	Charge model 8	168.19	4.5
Isomaltotriose			
2	Charge model 9	204.34	2.84
2	Charge model 10	197.39	0.00
Lactose			
3	Charge model 1	173.41	0.00
	Charge model 4	173.76	10.39
Lactulose			
4	Charge model 3	173.39	0.00
	Charge model 8	173.21	0.00
Maltose			
5	Charge model 1	171.74	0.00
	Charge model 3	174.55	0.88
	Charge model 8	174.14	0.87
Mannotetraose			
6	Charge model 1	278.55	6.87
	Charge model 3	272.34	7.42
	Charge model 7	259.88	0.00
	Charge model 10	261.77	4.68
Melibiose			
7	Charge model 1	172.88	0.00
	Charge model 3	176.78	8.07
	Charge model 5	169.40	9.20
Palatinose			
8	Charge model 1	164.42	2.52
	Charge model 3	168.45	0.00
	Charge model 8	167.69	0.00
Tagatose			
9	Charge model 4	129.87	4.41
	Charge model 5	126.99	0.00
Xylobiose			
10	Charge model 1	170.33	9.81
	Charge model 5	167.29	0.00
Cellobiose			
11	Charge model 1	170.45	0.00
	Charge model 8	168.84	0.61
Lacto-N-Fucopentaose I			
12	Charge model 4	312.77	0.00
	Charge model 7	312.80	0.00
	Charge model 10	317.99	5.77

Lacto-N-Neotetraose			
13	Charge model 4	277.22	0.01
	Charge model 7	277.93	0.00
	Charge model 14	278.56	2.00
Maltotetraose			
14	Charge model 6	252.44	2.56
	Charge model 8	248.27	7.15
	Charge model 9	249.01	1.71
	Charge model 14	254.71	0.00
Mannohexaose			
15	Charge model 10	362.16	4.30
	Charge model 13	363.03	0.00
	Charge model 16	363.11	6.93
	Charge model 20	374.76	5.07
Melezitose			
16	Charge model 1	211.55	3.63
	Charge model 2	230.54	5.20
	Charge model 3	205.97	0.00
	Charge model 9	212.82	1.37
	Charge model 10	201.54	0.52
Xylitol			
17	Charge model 1	123.77	0.00
	Charge model 2	123.68	0.00
	Charge model 4	123.38	0.00
Sorbitol			
18	Charge model 1	134.38	0.00
	Charge model 2	134.44	0.00
	Charge model 4	130.76	0.46
Inositol			
19	Charge model 1	130.75	2.69
	Charge model 2	129.91	0.00
	Charge model 3	130.06	4.17
	Charge model 6	129.43	4.96
Glucuronic acid			
20	Charge model 5	133.72	0.00
Sucrose			
21	Charge model 1	173.98	6.82
	Charge model 2	173.95	6.82
	Charge model 6	174.22	6.81
	Charge model 8	167.80	0.00
Mannitol			
22	Charge model 3	127.61	4.05
	Charge model 4	127.40	0.00
	Charge model 5	127.48	0.00

Raffinose			
23	Charge model 8	209.15	0.00
	Charge model 9	207.03	3.71

Figure S1. The final representative charge states for the 27 glycan systems used in this work.

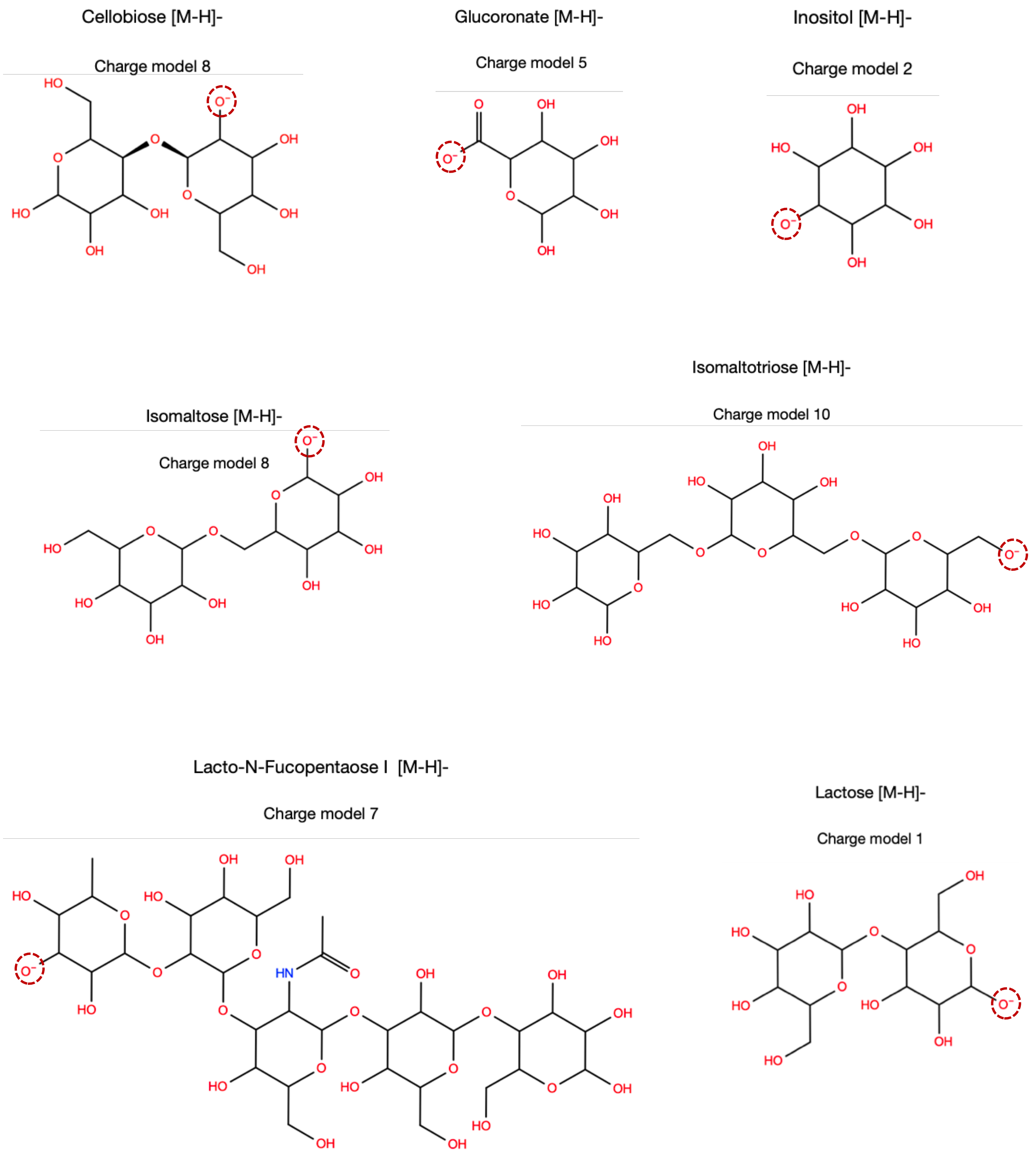

Lacto-N-Neotetraose [M-H]-

Charge model 7

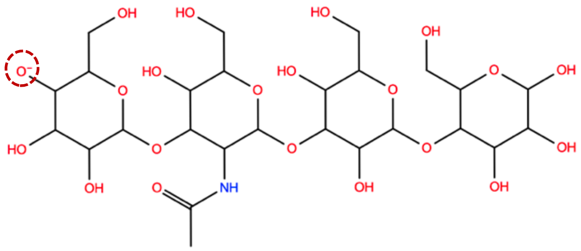

Lactulose [M-H]-

Charge model 7

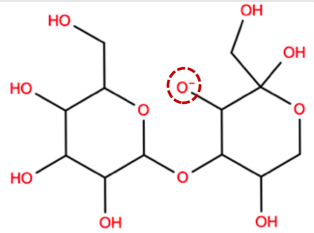

Maltose [M-H]-

Charge model 8

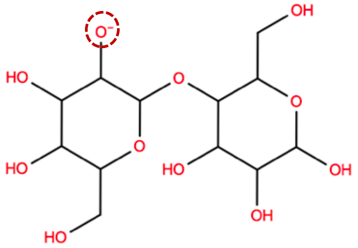

Maltotetraose [M-H]-

Charge model 6

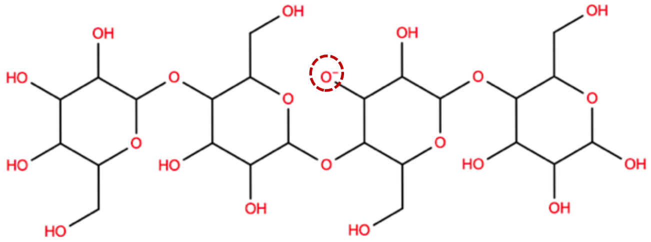

Mannotetraose [M-H]-

Charge model 3

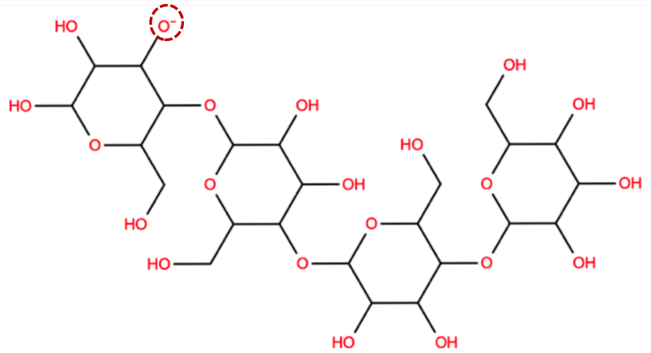

Mannitol [M-H]-

Charge model 4

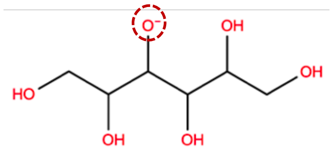

Melezitose [M-H]-

Charge model 1

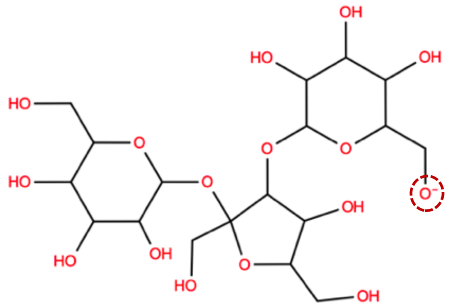

Melibiose [M-H]-

Charge model 1

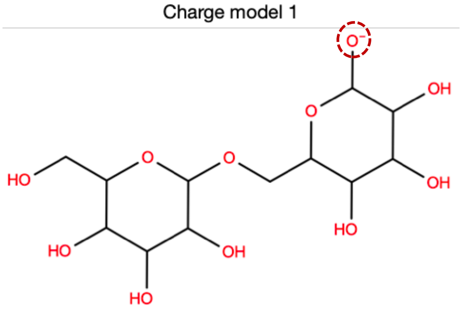

Raffinose [M-H]-

Charge model 9

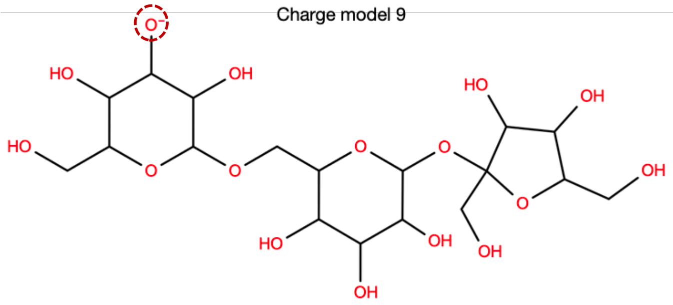

Xylitol [M-H]-

Charge model 2

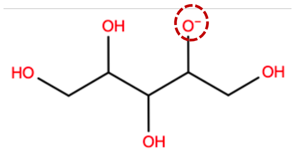

Palatinose [M-H]-

Charge model1

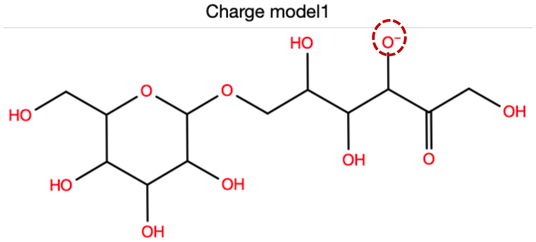

Sorbitol [M-H]-

Charge model 1

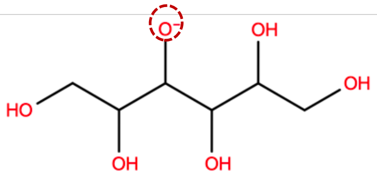

Stachyose [M-H]-

Charge model 9

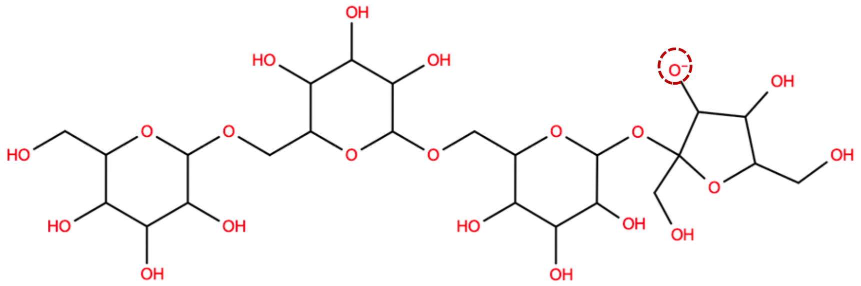

Tagatose [M-H]-

Charge model 5

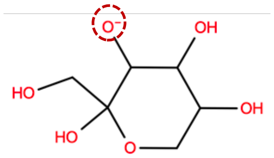

Sucrose [M-H]-

Charge model 1

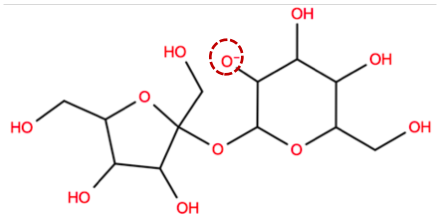

Xylobiose [M-H]-

Charge model 1

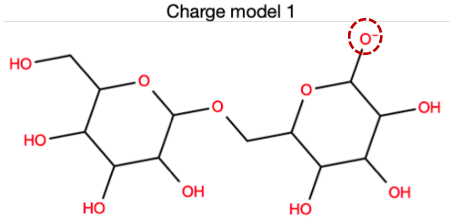

Melezitose [M+H]+

Charge model 7

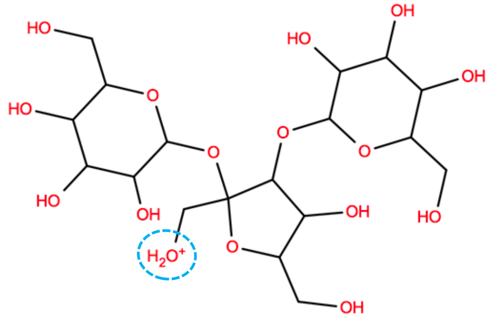

Sorbitol [M+H]+

Charge model 3

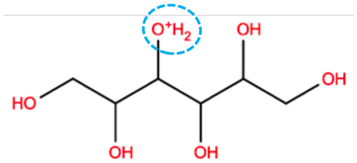

Glucosamine [M+H]+

Charge model 2

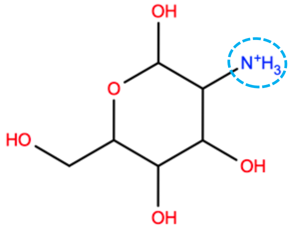

Maltotetraose [M+H]+

Charge model 7

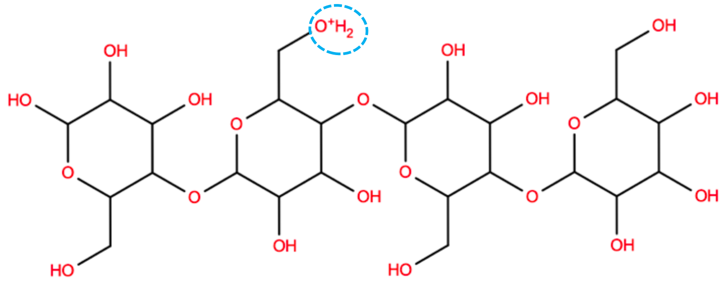

Table S3. DFT relative energies and computed CCS results for the final [M-H]$^-$ and [M+H]$^+$ charge model candidates. The mole fraction is the percentage of conformer(s) populated in gas phase for a system according to a Boltzmann-weighted average as a function of conformer relative energy across all charge models. Any conformer with RE that is within 3 kcal mol$^-$ of a global minimum conformer populates >0%.

Cellobiose Model 8 [M-H]-	Conformer #	Calculated CCS	Relative Energy (kcal mol-1)	Mol Fraction
1	34	171.7448	4.54	0.00
2	112	172.7234	13.65	0.00
3	123	174.0787	2.69	0.01
4	197	179.6718	1.40	0.08
5	234	173.2966	13.71	0.00
6	448	172.1120	12.30	0.00
7	472	180.7172	1.99	0.03
8	474	174.0034	0.00	0.85
9	535	163.4159	1.97	0.03
10	549	171.4934	5.57	0.00
11	584	169.9649	5.11	0.00
12	727	165.5134	6.11	0.00
13	746	176.0475	5.09	0.00
14	879	171.1035	15.27	0.00
15	883	171.9679	12.85	0.00
16	966	172.8099	11.71	0.00

Glucoronate Model 5 [M-H]-	Conformer #	Calculated CCS	Relative Energy (kcal mol-1)	Mol Fraction
1	7	132.9828	8.23	0.00
2	34	134.3425	6.22	0.00
3	37	133.4931	8.22	0.00
4	60	128.3007	0.00	0.50
5	81	128.5144	0.00	0.50

Inositol Model 2 [M-H]-	Conformer #	Calculated CCS	Relative Energy (kcal mol-1)	Mol Fraction
1	1	129.9077	0.00	0.85
2	2	129.8097	13.13	0.00
3	3	129.9462	13.13	0.00
4	4	129.8234	13.13	0.00
5	5	130.1444	10.38	0.00
6	6	130.0749	31.10	0.00
7	7	129.6306	28.30	0.00
8	8	129.794	28.30	0.00
9	9	128.9363	1.05	0.15
10	10	130.1428	10.38	0.00

11	11	129.7911	13.13	0.00
12	12	129.9352	13.13	0.00
13	13	129.9338	13.13	0.00
14	14	129.9975	10.38	0.00
15	15	129.9373	10.38	0.00
16	16	130.2331	10.38	0.00
17	17	129.9753	10.38	0.00
18	18	130.0996	10.38	0.00
19	19	130.037	10.38	0.00
20	20	130.1008	10.38	0.00
21	21	130.1092	10.38	0.00
22	22	129.9806	10.38	0.00
23	23	129.9814	10.38	0.00
24	24	128.4649	0.00	0.85
25	25	128.464	0.00	0.85
26	26	128.4943	0.00	0.85
27	27	128.5674	0.00	0.85
28	28	129.063	0.00	0.85
29	29	128.6438	0.00	0.85
30	30	128.8206	1.05	0.15
31	31	128.7438	0.00	0.86
32	32	128.7112	0.00	0.85
33	33	128.6673	1.05	0.15
34	34	125.5039	1.97	0.03

**Isomaltose Model 8 [M-H]-**	**Conformer #**	**Calculated CCS**	**Relative Energy (kcal mol-1)**	**Mol Fraction**
1	14	176.5267	7.34	0.00
2	34	168.7328	5.74	0.00
3	110	171.1706	9.11	0.00
4	123	176.8444	6.04	0.00
5	197	175.308	19.31	0.00
6	234	180.9502	13.54	0.00
7	242	167.3077	0.89	0.17
8	315	170.6027	0.00	0.78
9	448	166.6643	8.54	0.00
10	472	172.2367	1.71	0.04
11	617	164.0726	2.80	0.01
12	669	185.0079	32.04	0.00
13	772	166.8572	15.98	0.00
14	879	164.7374	14.43	0.00
15	883	166.0072	30.13	0.00
16	962	172.1341	22.16	0.00
17	966	165.1254	13.79	0.00

Isomaltotriose Model 10 [M-H]-	Conformer #	Calculated CCS	Relative Energy (kcal mol-1)	Mol Fraction
1	102	230.4365	42.91	0.00
2	110	232.1271	42.29	0.00
3	123	212.3339	6.88	0.00
4	137	228.8477	30.37	0.00
5	14	205.5229	24.59	0.00
6	196	208.2935	16.81	0.00
7	197	224.7799	26.98	0.00
8	231	223.1062	29.42	0.00
9	234	216.4835	36.79	0.00
10	304	222.2571	44.65	0.00
11	306	220.4394	41.08	0.00
12	307	212.7994	25.57	0.00
13	315	223.5059	26.15	0.00
14	34	216.1035	26.03	0.00
15	364	229.3775	25.77	0.00
16	382	202.7129	9.88	0.00
17	446	212.9277	27.02	0.00
18	472	223.306	38.65	0.00
19	474	209.1621	46.42	0.00
20	497	207.9848	2.13	0.03
21	549	211.2927	35.59	0.00
22	585	213.4731	22.96	0.00
23	669	227.0575	39.32	0.00
24	746	225.3219	41.20	0.00
25	771	218.3516	33.84	0.00
26	797	215.3912	33.15	0.00
27	807	222.6898	41.38	0.00
28	828	213.313	36.99	0.00
29	841	209.9075	35.11	0.00
30	879	227.8907	45.09	0.00
31	883	207.9855	0.00	0.97
32	898	216.237	41.40	0.00
33	961	213.4588	18.27	0.00
34	962	231.9075	41.73	0.00

LNF1 Model 7 [M-H]-	Conformer #	Calculated CCS	Relative Energy (kcal mol-1)	Mol Fraction
1	34	290.1736	25.63	0.00
2	42	293.0578	12.52	0.00
3	84	309.1181	30.77	0.00
4	95	323.5442	10.72	0.00
5	99	312.7489	17.18	0.00
6	102	321.6982	6.62	0.00
7	105	321.6668	19.67	0.00
8	112	320.7156	36.30	0.00
9	123	305.9749	41.47	0.00
10	126	330.6259	34.30	0.00
11	153	320.1506	17.80	0.00
12	156	318.6847	6.37	0.00
13	217	308.0872	41.95	0.00
14	219	304.3347	7.68	0.00
15	234	295.351	0.00	1.00
16	304	311.3795	6.02	0.00
17	306	301.3089	18.58	0.00
18	472	324.2197	30.25	0.00
19	474	299.0683	17.43	0.00
20	497	296.6264	12.98	0.00

LNN Model 7 [M-H]-	Conformer #	Calculated CCS	Relative Energy (kcal mol-1)	Mol Fraction
1	14	267.7973	28.83	0.00
2	34	284.9761	27.13	0.00
3	102	279.8534	34.33	0.00
4	112	277.8462	29.24	0.00
5	115	272.8949	38.62	0.00
6	153	283.4675	29.52	0.00
7	156	283.1206	48.51	0.00
8	234	284.3603	19.78	0.00
9	236	282.246	43.14	0.00
10	242	272.6885	38.61	0.00
11	304	286.0103	44.63	0.00
12	364	277.9417	22.50	0.00
13	365	256.6083	26.49	0.00
14	367	283.4265	39.49	0.00
15	403	289.0674	31.37	0.00
16	430	286.4293	25.30	0.00
17	446	281.4686	23.94	0.00
18	471	281.0535	23.63	0.00
19	472	277.9849	349.64	0.00
20	578	271.2615	59.03	0.00
21	610	251.9559	0.00	1.00

22	669	271.5134	21.08	0.00
23	684	291.3737	27.06	0.00
24	746	261.597	31.87	0.00
25	771	280.7775	53.94	0.00
26	856	279.6269	63.85	0.00
27	879	280.8259	34.05	0.00
28	883	253.4667	40.67	0.00
29	962	265.3128	25.76	0.00
30	981	280.1032	49.26	0.00

Lactose Model 1 [M-H]-	Conformer #	Calculated CCS	Relative Energy (kcal mol-1)	Mol Fraction
1	123	180.2273	11.14	0.00
2	231	174.1787	2.26	0.02
3	234	176.6116	11.88	0.00
4	448	168.4804	10.59	0.00
5	472	170.3669	10.72	0.00
6	535	184.0852	9.94	0.00
7	549	171.4553	0.00	0.98
8	684	178.1771	14.97	0.00
9	746	179.0662	11.89	0.00
10	771	174.7287	10.63	0.00
11	879	178.4852	6.91	0.00
12	962	176.4955	13.67	0.00

Lactulose Model 8 [M-H]-	Conformer #	Calculated CCS	Relative Energy (kcal mol-1)	Mol Fraction
1	14	174.0337	16.86	0.00
2	102	173.4533	14.19	0.00
3	123	174.1131	8.19	0.00
4	126	173.3782	15.55	0.00
5	304	177.3184	4.27	0.00
6	306	175.9703	0.00	0.63
7	367	167.0913	32.60	0.00
8	448	177.4446	7.72	0.00
9	472	168.582	9.58	0.00
10	535	176.7217	18.38	0.00
11	684	176.38	0.33	0.36
12	727	181.3438	24.86	0.00
13	771	172.2676	8.01	0.00
14	879	165.8956	12.96	0.00
15	883	175.1635	3.01	0.00
16	912	172.9889	8.97	0.00
17	962	181.8342	38.22	0.00
18	984	169.3047	9.59	0.00

Maltose Model 8 [M-H]-	Conformer #	Calculated CCS	Relative Energy (kcal mol-1)	Mol Fraction
1	34	168.3948	13.89	0.00
2	102	176.2972	0.82	0.19
3	123	163.6572	16.22	0.00
4	156	170.6121	5.83	0.00
5	217	174.203	6.47	0.00
6	234	170.2706	12.72	0.00
7	448	176.8704	17.04	0.00
8	472	175.8425	0.00	0.74
9	549	166.3929	20.52	0.00
10	607	167.8952	12.55	0.00
11	684	166.2906	1.34	0.08
12	771	177.2229	15.93	0.00
13	772	164.7449	7.34	0.00
14	879	166.5079	8.51	0.00
15	883	184.3276	21.00	0.00
16	950	165.3041	7.90	0.00
17	961	169.4488	15.80	0.00

Maltotetraose Model 6 [M-H]-	Conformer #	Calculated CCS	Relative Energy (kcal mol-1)	Mol Fraction
1	14	253.768	0.00	0.96
2	34	251.407	5.30	0.00
3	102	249.7764	8.58	0.00
4	110	244.7446	5.19	0.00
5	123	259.3489	16.67	0.00
6	126	256.2887	20.58	0.00
7	156	265.8308	20.51	0.00
8	197	249.4628	11.02	0.00
9	234	269.8272	27.70	0.00
10	304	256.4018	8.39	0.00
11	315	239.5078	20.88	0.00
12	355	255.4925	3.49	0.00
13	448	264.6565	1.92	0.04
14	469	242.7732	17.88	0.00
15	472	259.5565	16.83	0.00
16	474	247.5886	12.52	0.00
17	549	269.8804	25.11	0.00
18	669	242.319	34.48	0.00
19	684	248.2576	29.50	0.00
20	771	246.5279	20.76	0.00
21	797	243.0358	33.06	0.00
22	879	253.7329	31.68	0.00
23	883	262.9222	39.65	0.00
24	912	247.0716	19.36	0.00

25	966	251.5764	36.34	0.00
26	978	255.0958	44.29	0.00

Maltotetraose Model 6 [M-H]-	Conformer #	Calculated CCS	Relative Energy (kcal mol-1)	Mol Fraction
1	34	126.2134	2.55	0.01
2	102	128.242	4.97	0.00
3	234	131.3729	15.39	0.00
4	304	127.7832	9.09	0.00
5	472	127.7721	3.68	0.00
6	549	131.0233	20.38	0.00
7	586	128.711	8.30	0.00
8	669	129.0323	12.58	0.00
9	727	130.1742	13.99	0.00
10	746	127.7978	0.00	0.56
11	879	127.1424	2.17	0.01
12	883	126.2767	2.17	0.01
13*	797	128.2138	0.62	0.20
14*	234	128.2894	0.62	0.20

*Model 5 conformer

Mannotetraose Model 3 [M-H]-	Conformer #	Calculated CCS	Relative Energy (kcal mol-1)	Mol Fraction
1	34	239.8556	2.84	0.01
2	60	244.1071	18.09	0.00
3	95	257.2105	9.37	0.00
4	112	292.673	37.05	0.00
5	123	273.707	26.74	0.00
6	126	281.5038	31.57	0.00
7	153	270.7345	32.90	0.00
8	156	271.0459	26.41	0.00
9	205	244.8948	17.64	0.00
10	211	275.2866	38.54	0.00
11	231	251.9432	28.76	0.00
12	234	252.3073	27.02	0.00
13	306	259.0662	19.78	0.00
14	308	276.5911	45.37	0.00
15	353	238.5171	0.00	0.99
16	448	272.6995	25.15	0.00
17	549	275.5916	31.13	0.00
18	584	270.5584	24.15	0.00
19	625	256.4076	3.87	0.00
20	662	258.9165	21.55	0.00
21	669	276.7943	46.49	0.00
22	746	276.4752	34.48	0.00

23	772	267.9605	26.00	0.00
24	876	276.2337	33.81	0.00
25	883	272.8993	22.35	0.00
26	966	270.704	111.73	0.00

Melezitose Model 1 [M-H]-	Conformer #	Calculated CCS	Relative Energy (kcal mol-1)	Mol Fraction
1	34	191.9581	8.69	20.00
2	84	201.6196	3.21	0.00
3	102	208.6783	4.10	0.00
4	123	212.5482	25.40	0.00
5	137	201.7472	0.00	0.99
6	156	197.7639	15.24	0.00
7	204	201.6491	18.97	0.00
8	234	212.9616	21.18	0.00
9	242	203.7251	16.99	0.00
10	472	215.6903	13.89	0.00
11	474	208.0916	22.43	0.00
12	497	204.7588	4.40	0.00
13	535	209.2268	33.90	0.00
14	549	219.5698	28.40	0.00
15	669	209.7622	28.61	0.00
16	684	213.7033	23.64	0.00
17	705	222.5326	23.48	0.00
18	727	203.6152	8.51	0.00
19	807	202.7703	22.70	0.00
20	879	204.8422	14.09	0.00
21	883	207.6198	25.11	0.00
22	948	200.0647	14.98	0.00
23	950	198.5731	6.41	0.00
24	962	195.2319	1.33	0.11

Melibiose Model 1 [M-H]-	Conformer #	Calculated CCS	Relative Energy (kcal mol-1)	Mol Fraction
1	14	164.7815	0.00	1.00
2	34	181.7824	14.64	0.00
3	112	177.9933	12.36	0.00
4	123	173.9483	13.73	0.00
5	156	174.6024	28.78	0.00
6	234	174.1043	6.59	0.00
7	353	181.955	21.86	0.00
8	448	176.9512	20.16	0.00
9	472	169.1747	17.17	0.00
10	474	175.0823	28.64	0.00

11	497	182.9742	35.15	0.00
12	549	165.0389	17.88	0.00
13	669	162.8084	19.49	0.00
14	727	170.3973	11.20	0.00
15	797	185.5947	31.23	0.00
16	879	166.57	11.97	0.00
17	883	174.3176	17.61	0.00

Palatinose Model 1 [M-H]-	Conformer #	Calculated CCS	Relative Energy (kcal mol-1)	Mol Fraction
1	14	173.4409	14.29	0.00
2	34	160.4758	13.37	0.00
3	156	174.3832	10.04	0.00
4	197	175.4132	24.52	0.00
5	234	168.0916	16.39	0.00
6	472	171.7321	29.45	0.00
7	474	172.4112	9.79	0.00
8	549	163.5013	13.96	0.00
9	608	171.549	9.44	0.00
10	669	159.1039	8.60	0.00
11	684	175.9827	0.00	0.56
12	772	166.2111	24.90	0.00
13	876	172.2865	14.63	0.00
14	879	168.2158	10.62	0.00
15	883	176.0736	14.87	0.00
16*	102	165.1934	0.14	0.44

*Model 3 conformer

Raffinose Model 9 [M-H]-	Conformer #	Calculated CCS	Relative Energy (kcal mol-1)	Mol Fraction
1	12	226.3643	34.83	0.00
2	14	217.6408	27.09	0.00
3	34	194.9663	0.00	0.82
4	95	217.7078	9.19	0.00
5	112	207.46	4.58	0.00
6	121	222.6302	24.34	0.00
7	123	208.5741	15.30	0.00
8	231	214.0734	17.69	0.00
9	234	207.6389	8.56	0.00
10	353	215.824	16.52	0.00
11	357	217.5176	24.39	0.00
12	399	207.0606	13.07	0.00
13	448	218.3334	26.56	0.00
14	472	224.0585	21.14	0.00
15	474	206.6056	25.49	0.00

16	497	205.9726	10.67	0.00
17	515	217.4236	20.14	0.00
18	549	216.9728	19.48	0.00
19	578	226.7486	30.81	0.00
20	584	224.6682	20.99	0.00
21	684	218.9835	13.48	0.00
22	727	223.7338	17.90	0.00
23	731	203.63	17.38	0.00
24	772	219.8294	34.23	0.00
25	796	215.6203	19.91	0.00
26	841	203.2074	11.89	0.00
27	863	222.6602	30.34	0.00
28	879	218.5019	19.34	0.00
29	883	202.0409	16.68	0.00
30	962	232.241	36.08	0.00
31	966	203.7582	16.10	0.00
32*	705	215.3734	0.91	0.18

*Model 8 conformer

Sophorose Model 1 [M-H]-	Conformer #	Calculated CCS	Relative Energy (kcal mol-1)	Mol Fraction
1	34	183.0138	16.19	0.00
2	123	166.7477	0.00	1.00
3	231	174.4917	17.05	0.00
4	234	175.5505	4.63	0.00
5	242	182.4301	15.59	0.00
6	306	176.6319	9.23	0.00
7	472	167.2002	5.97	0.00
8	474	164.6489	16.24	0.00
9	549	183.5517	24.15	0.00
10	674	176.178	21.67	0.00
11	684	179.97	23.48	0.00
12	746	164.6837	3.84	0.00
13	879	163.2563	6.91	0.00
14	950	172.9933	24.70	0.00
15	962	183.9974	22.57	0.00

Sorbitol Model 1 [M-H]-	Conformer #	Calculated CCS	Relative Energy (kcal mol-1)	Mol Fraction
1	14	129.8776	5.98	0.00
2	34	127.1765	3.57	0.00
3	234	130.062	11.77	0.00
4	472	130.5734	10.34	0.00
5	549	141.7405	30.42	0.00
6	684	127.8808	9.59	0.00

7	746	131.6765	14.69	0.00
8	879	128.3597	3.70	0.00
9	883	127.564	12.82	0.00
10	950	129.31	9.64	0.00
11	962	127.4369	14.78	0.00
12	966	124.3938	0.00	1.00

Sucrose Model 1 [M-H]-	Conformer #	Calculated CCS	Relative Energy (kcal mol-1)	Mol Fraction
1	34	172.8979	21.92	0.00
2	35	177.368	9.04	0.00
3	102	176.2956	27.83	0.00
4	121	169.5447	12.26	0.00
5	123	170.7328	0.00	0.98
6	156	170.8462	9.42	0.00
7	231	166.9301	4.25	0.00
8	234	180.2265	28.47	0.00
9	304	172.0585	6.24	0.00
10	472	171.3432	7.43	0.00
11	538	170.915	12.26	0.00
12	549	169.7777	2.42	0.02
13	669	174.96	16.71	0.00
14	684	177.6829	21.77	0.00
15	876	165.0339	6.80	0.00
16	879	175.072	10.86	0.00
17	950	171.4913	13.02	0.00
18	997	167.7086	15.09	0.00

Tagatose Model 5 [M-H]-	Conformer #	Calculated CCS	Relative Energy (kcal mol-1)	Mol Fraction
1	1	131.4297	26.81	0.00
2	2	131.4329	26.81	0.00
3	3	128.6906	8.91	0.00
4	4	126.7908	1.75	0.00
5	5	127.0064	1.75	0.00
6	6	128.7967	8.91	0.00
7	7	128.8322	8.91	0.00
8	8	126.5906	1.75	0.00
9	9	126.7977	1.75	0.00
10	10	126.3717	0.54	0.04
11	11	126.2788	0.54	0.04
12	12	126.5801	0.54	0.04
13	13	126.3047	0.54	0.04
14	14	127.5581	2.38	0.00

15	15	127.6998	2.38	0.00
16	16	128.5735	10.96	0.00
17	17	128.6941	10.96	0.00
18	18	127.3176	2.38	0.00
19	19	127.572	2.38	0.00
20	20	128.9102	10.96	0.00
21	21	128.5702	10.96	0.00
22	22	126.084	0.00	0.09
23	23	125.9931	0.00	0.10
24	24	126.1215	0.00	0.10
25	25	126.1324	0.00	0.10
26	26	131.4982	26.05	0.00
27	27	128.6571	8.62	0.00
28	28	128.7393	8.62	0.00
29	29	128.6991	8.62	0.00
30	30	128.5509	10.97	0.00
31	31	128.7138	10.96	0.00
32	32	128.5767	10.96	0.00
33	33	128.9959	8.62	0.00
34	34	127.6562	2.38	0.00
35	35	127.671	2.38	0.00
36	36	127.2504	2.38	0.00
37	37	127.4192	2.38	0.00
38	38	129.0419	18.96	0.00
39	39	127.0711	1.75	0.00
40	40	126.6767	1.75	0.00
41	41	126.9146	1.75	0.00
42	42	128.713	16.17	0.00
43	43	126.5158	0.54	0.04
44	45	126.3071	0.54	0.04
45	46	126.0732	0.00	0.09
46	47	125.9588	0.00	0.10
47	48	126.1033	0.00	0.10
48	49	126.0753	0.00	0.10
49	60	128.1271	9.10	0.00
50	76	128.2293	9.10	0.00

Xylitol Model 2 [M-H]-	Conformer #	Calculated CCS	Relative Energy (kcal mol-1)	Mol Fraction
1	34	121.1756	0.00	0.50
2	112	121.8716	2.83	0.00
3	234	123.8994	4.67	0.00
4	472	122.4414	16.88	0.00
5	549	121.3043	0.00	0.50
6	669	122.256	3.20	0.00

7	684	120.6566	5.62	0.00

Xylobiose Model 1 [M-H]-	Conformer #	Calculated CCS	Relative Energy (kcal mol-1)	Mol Fraction
1	34	183.0507	16.19	0.00
2	123	166.7477	0.00	1.00
3	231	174.4917	17.05	0.00
4	234	175.4868	4.63	0.00
5	242	182.4126	15.59	0.00
6	306	176.6566	9.23	0.00
7	472	167.2335	5.97	0.00
8	474	164.6489	16.24	0.00
9	497	164.2901	9.01	0.00
10	549	183.5517	24.15	0.00
11	674	176.178	21.67	0.00
12	684	179.9284	23.48	0.00
13	746	164.6419	3.84	0.00
14	879	163.3443	6.91	0.00
15	950	172.891	24.70	0.00
16	962	183.9974	22.57	0.00

Glucosamine Model 2 [M+H]+	Conformer #	Calculated CCS	Relative Energy (kcal mol-1)	Mol Fraction
1	2	132.8742	6.81	0.00
2	3	132.7488	6.81	0.00
3	4	132.3371	5.59	0.00
4	5	133.4415	9.62	0.00
5	6	131.8332	5.82	0.00
6	7	129.7281	2.38	0.00
7	8	131.9437	5.82	0.00
8	9	129.7467	2.38	0.00
9	10	129.357	0.00	0.25
10	11	129.2405	0.00	0.25
11	12	129.2321	0.00	0.25
12	13	129.2423	0.00	0.25
13	14	133.4605	6.45	0.00
14	15	133.4752	6.45	0.00
15	16	132.5379	6.81	0.00
16	17	132.6079	6.81	0.00
17	18	133.3204	4.95	0.00
18	19	133.4182	4.95	0.00
19	20	132.595	5.59	0.00
20	21	132.6824	5.59	0.00
21	22	131.918	5.82	0.00

22	23	129.6113	2.38	0.00
23	24	131.9373	5.82	0.00
24	25	129.6884	2.38	0.00
25	26	129.3875	0.00	0.25
26	27	129.2552	0.00	0.25
27	28	129.3343	0.00	0.25
28	29	129.1902	0.00	0.25
29	30	133.4137	6.45	0.00
30	31	133.5032	6.45	0.00
31	32	133.3129	4.95	0.00
32	33	134.2117	9.23	0.00
33	34	132.2153	5.21	0.00
34	35	132.2174	6.92	0.00
35	36	132.2102	5.21	0.00
36	37	131.2002	2.12	0.01
37	38	132.4969	5.21	0.00
38	39	131.025	2.12	0.01
39	40	132.474	5.21	0.00
40	41	131.3222	2.12	0.01
41	42	130.3853	0.53	0.10
42	43	130.236	0.53	0.10
43	44	129.966	0.53	0.10
44	45	130.0623	0.53	0.10
45	46	129.555	0.00	0.25
46	47	129.2878	0.00	0.25
47	48	129.3651	0.00	0.25
48	49	129.3851	0.00	0.25
49	60	129.6267	3.01	0.00
50	74	129.5134	3.01	0.00

Maltotetraose Model 7 [M+H]+	Conformer #	Calculated CCS	Relative Energy (kcal mol-1)	Mol Fraction
1	34	254.5097	39.12	0.00
2	102	259.0155	16.64	0.00
3	112	252.9964	10.11	0.00
4	123	256.0136	18.66	0.00
5	234	242.9353	14.94	0.00
6	239	254.3966	15.42	0.00
7	304	247.1635	5.50	0.00
8	446	261.2887	34.82	0.00
9	448	251.2016	17.74	0.00
10	472	262.5906	20.84	0.00
11	474	252.2693	5.00	0.00
12	497	257.0983	13.75	0.00
13	529	251.1342	23.26	0.00

14	684	236.5075	0.00	1.00
15	727	261.1714	21.23	0.00
16	796	238.7021	15.91	0.00
17	879	231.4139	0.82	0.25
18	883	248.8638	10.67	0.00
19	961	256.3857	24.34	0.00
20	962	250.966	18.47	0.00
21	966	260.8742	32.80	0.00

Melezitose Model 7 [M+H]+	Conformer #	Calculated CCS	Relative Energy (kcal mol-1)	Mol Fraction
1	34	204.33	31.86	0.00
2	81	202.6524	22.75	0.00
3	102	193.4354	0.10	0.46
4	126	211.6388	7.49	0.00
5	234	207.183	23.23	0.00
6	306	211.4345	23.90	0.00
7	370	197.2977	11.89	0.00
8	472	206.5143	0.00	0.54
9	549	204.0841	28.08	0.00
10	578	212.2186	31.31	0.00
11	625	202.9248	19.05	0.00
12	669	195.1528	9.74	0.00
13	746	206.8001	28.25	0.00
14	879	213.2803	34.50	0.00
15	883	204.2365	6.41	0.00
16	950	208.2228	30.41	0.00
17	961	203.4023	4.17	0.00
18	962	203.3236	13.34	0.00

Sorbitol Model 3 [M+H]+	Conformer #	Calculated CCS	Relative Energy (kcal mol-1)	Mol Fraction
1	34	128.2822	0.00	0.41
2	102	126.8668	0.79	0.11
3	123	127.3132	7.56	0.00
4	472	130.9377	0.19	0.30
5	549	129.8416	9.62	0.00
6	727	127.4217	0.46	0.19
7	746	133.8847	7.85	0.00
8	879	129.4371	6.70	0.00
9	950	134.4766	20.76	0.00
10	961	134.6446	20.59	0.00
11	962	130.6802	11.30	0.00

Table S4. The molecular surface area measurments for α and β anomers. The % MSA Diff. is the percent difference be between the α and β MSA values. The structures were build through the Glycam webserver.

Anomer Pair	α Sequence	α MSA (Å²)	β Sequence	β MSA (Å²)	% MSA Diff.
1	DManpa1-4DManpa1-OH	356.00	DManpb1-4DManpa1-OH	360.26	1.19
2	DGalpa1-4DManpa1-OH	347.66	DGalpb1-4DManpa1-OH	358.53	3.08
3	DGlcpa1-4DManpa1-OH	354.21	DGlcpb1-4DManpa1-OH	358.53	1.21
4	DAllpa1-4DManpa1-OH	344.07	DAllpb1-4DManpa1-OH	357.06	3.71
5	DAltpa1-4DManpa1-OH	349.83	DAltpb1-4DManpa1-OH	358.78	2.53
6	DGulpa1-4DManpa1-OH	347.12	DGulpb1-4DManpa1-OH	359.83	3.60
7	DTalpa1-4DManpa1-OH	349.08	DTalpb1-4DManpa1-OH	356.31	2.05
8	DRibpa1-4DManpa1-OH	319.75	DRibpb1-4DManpa1-OH	328.10	2.58
9	DFrupa2-4DManpa1-OH	335.67	DFrupb2-4DManpa1-OH	335.85	0.05
10	DGlcpNAca1-4DManpa1-OH	399.01	DGlcpNAcb1-4DManpa1-OH	410.44	2.83
11	DManpa1-4DGlcpa1-OH	353.70	DManpb1-4DGlcpa1-OH	358.01	1.21
12	DGalpa1-4DGlcpa1-OH	346.43	DGalpb1-4DGlcpa1-OH	356.18	2.77
13	DGlcpa1-4DGlcpa1-OH	351.91	DGlcpb1-4DGlcpa1-OH	355.71	1.08
14	DAllpa1-4DGlcpa1-OH	344.89	DAllpb1-4DGlcpa1-OH	354.71	2.81
15	DAltpa1-4DGlcpa1-OH	347.76	DAltpb1-4DGlcpa1-OH	355.98	2.33
16	DTalpa1-4DGlcpa1-OH	346.29	DTalpb1-4DGlcpa1-OH	353.73	2.12
17	DRibpa1-4DGlcpa1-OH	319.01	DRibpb1-4DGlcpa1-OH	325.69	2.07
18	DFrupa2-4DGlcpa1-OH	333.91	DFrupb2-4DGlcpa1-OH	333.11	0.24
19	DGlcpNAca1-4DGlcpa1-OH	396.36	DGlcpNAcb1-4DGlcpa1-OH	407.83	2.85
20	DManpa1-4DRibpa1-OH	321.53	DManpb1-4DRibpa1-OH	325.93	1.36
21	DGalpa1-4DRibpa1-OH	317.43	DGalpb1-4DRibpa1-OH	323.84	2.00
22	DGlcpa1-4DRibpa1-OH	320.03	DGlcpb1-4DRibpa1-OH	324.00	1.23
23	DAllpa1-4DRibpa1-OH	319.24	DAllpb1-4DRibpa1-OH	322.65	1.06
24	DAltpa1-4DRibpa1-OH	319.79	DAltpb1-4DRibpa1-OH	324.14	1.35
25	DGulpa1-4DRibpa1-OH	316.33	DGulpb1-4DRibpa1-OH	325.40	2.82
26	DTalpa1-4DRibpa1-OH	317.73	DTalpb1-4DRibpa1-OH	321.31	1.12
27	DRibpa1-4DRibpa1-OH	287.51	DRibpb1-4DRibpa1-OH	293.62	2.10
28	DFrupa2-4DRibpa1-OH	313.68	DFrupb2-4DRibpa1-OH	311.93	0.56
29	DGlcpNAca1-4DRibpa1-OH	364.31	DGlcpNAcb1-4DRibpa1-OH	375.68	3.07
30	DManpa1-4DFrupa2-OH	338.92	DManpb1-4DFrupa2-OH	354.74	4.56
31	DGlcpa1-4DFrupa2-OH	336.98	DGlcpb1-4DFrupa2-OH	353.20	4.70
32	DRibpa1-4DFrupa2-OH	310.58	DRibpb1-4DFrupa2-OH	323.08	3.95
33	DFrupa2-4DFrupa2-OH	336.79	DFrupb2-4DFrupa2-OH	336.63	0.05
34	DFrupa2-4DGlcpNAca1-OH	375.25	DFrupb2-4DGlcpNAca1-OH	374.75	0.13

35	DFrupa2-4DGalpa1-OH	330.79	DFrupb2-4DGalpa1-OH	336.44	1.69
36	DFrupa2-4DAllpa1-OH	336.01	DFrupb2-4DAllpa1-OH	333.60	0.72
	Average				2.02

Figure S2. Structures for six derivatized glycan isomers candidate charge models. Deprotonation sites obtained using D3BJ-B3LYP/6-31+G(d,p). For *isomers* 1, 2, 3, 4, and 5, the C2 hydroxyl of the terminal front monosaccharide residue is the favorable deprotonation site. The C3 hydroxyl for the internal galactose residue is deprotonated for isomer 6.

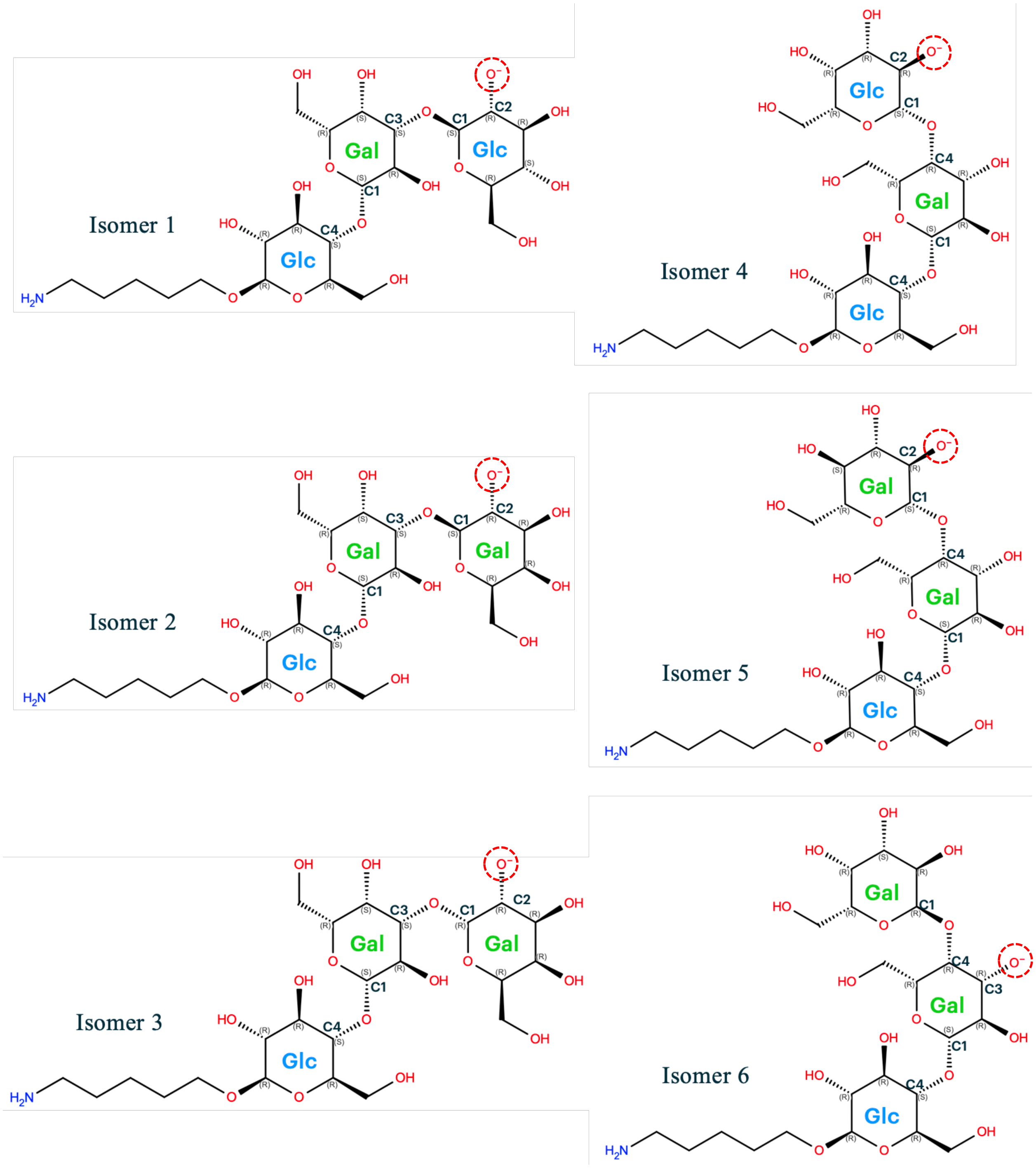

Table S5. Results after DFT optimization and energy screening step are the derivatized glycan candidate charge states with relative energy ≤10 kcal mol^{-1} of the minimum equilibrium structure for each.

Isomer	Charge State $[M-H]^-$	Cal. CCS ($Å^2$)	Relative Energy (kcal/mol)
1	Model 6	271.33	0.00
	Model 7	276.95	7.98
	Model 9	271.25	0.00
2	Model 6	273.78	0.64
	Model 7	282.38	8.06
	Model 9	273.03	0.00
3	Model 6	263.18	6.79
	Model 7	272.90	4.36
	Model 9	270.27	0.00
	Model 10	269.61	7.48
4	Model 7	273.75	1.58
	Model 9	273.12	0.00
5	Model 9	274.02	0.00
6	Model 3	267.05	9.99
	Model 6	268.71	7.83
	Model 7	268.06	0.00
